# Supplementary material for: Multiple Geographical Origins of Environmental Sex Determination enhanced the diversification of Darwin’s Favourite Orchids
Source: Sci Rep. 2017 Oct 10;7:12878. doi: 10.1038/s41598-017-12300-y (PMC5635016; doi:10.1038/s41598-017-12300-y)
Supplement: Supplementary file 1 — Supplementary Material [file 41598_2017_12300_MOESM1_ESM.pdf]

Multiple Geographical Origins of Environmental Sex Determination enhanced the diversification of Darwin's Favourite Orchids

Oscar Alejandro Pérez-Escobar[1*]; Guillaume Chomicki[2]; Fabien L. Condamine[3]; Jurriaan M. de Vos[4*]; Aline C. Martins[5]; Eric C. Smidt[5]; Bente Klitgård[1]; Günter Gerlach[6]; Jochen Heinrichs[2]

Appendix S1:

Including: Extended Materials and Methods.

Including: Supplementary tables (S1-S7) and figures (S1-S14).

Extended Materials and Methods

Phylogenetic incongruence analysis

PACo is a co-phylogenetic approach that is capable of assessing similarities between two trees by comparison of Euclidean distance matrices derived from phylogenies. Additionally, it determines the contribution of every association (i.e. pair of terminals of the analysed phylogenies) to the co-phylogenetic pattern observed, thus it has the potential to statistically identify specific potential conflicting terminals. PACo requires as input data a set of phylograms (e.g., posterior distribution of trees, or bootstrap replicate trees) derived from every genomic compartment (i.e. nucleus and plastids) and a binary association matrix, in which same taxa present in both datasets are linked. We executed PACo using 1000 trees from the Bayesian posterior distribution of trees obtained from MrBayes analysis computed from concatenated nuclear and plastid datasets, respectively. Plastids are maternally inherited in orchids[1,2], hence they reflect maternal evolutionary relationships between species. Contrastingly, nuclear loci show bi-parental inheritance[3], thus revealing maternal and paternal evolutionary histories – potentially revealing a more comprehensive perspective on the evolutionary relations between species. Therefore, conflicting plastid sequences were excluded from the dataset, and non-conflicting sequences of each locus were re-aligned and concatenated.

Ancestral area estimation (AAE)

We relied on BIOGEOBEARS (Biogeography with Bayesian and Likelihood Evolutionary Analysis in R script) to carry AAE analyses. Unlike previous programs such as LAGRANGE[4], 'BIOGEOBEARS' evaluates several biogeographical models altogether testing the role and contribution of evolutionary processes (i.e., range expansions, local extinctions, founder-event speciation, vicariance, and speciation in

the same biogeographic region) that were taken into account to explain extant, observed distributions in a joint statistical framework. Thus, it enables model testing, because distinct geographical and phylogenetic datasets can have biogeographic histories best modelled by different approaches[5]. We divided the geographical range of Catasetinae and outgroup taxa in eight biogeographic regions (1) Central America, comprising southern Mexico through Panama and the Antilles; (2) Guiana Shield, encompasses areas above 500 m in Colombia, Venezuela, Brazil, Guyana, Suriname and French Guiana; (3) Amazonia, encompassing lowlands and pre-montane forest below 800 m in Colombia, Ecuador, Peru, Brazil, Venezuela, Guyana, Suriname, and French Guiana[6]; (4) Chocó, comprising lowlands below 500 m of the western Andes in Colombia and Ecuador; (5) Northern Andes, includes elevated areas above 800 m from Southernmost Peru to Northern Colombia and Northeast Venezuela; (6) Central Andes (comprising areas above 800 m in Northern Peru to Northern Chile and Northeast Argentina); (7) South-eastern South America (encompassing part of the Brazilian shield, South-eastern Bolivia, Paraguay, Uruguay, and Northern Argentina); and (8) Africa and Australasia (representing distribution ranges of outgroup taxa).

Molecular clock dating

We selected the crown group of Catasetinae and the tree root (i.e., MRCA of Vandae +Cymbidieae), respectively. We set the tree root (i.e., MRCA of Vandae +Cymbidieae) to 34.05 ± 8 Myrs and the crown group of Catasetinae to 19.88 ± 6 Myrs, using a normal distribution. For strict molecular clock calibration, we placed a single constraint only at the tree root (34.05 ± 8 Myrs), using a normal distribution.

For each clock model, we ran two MCMC analyses with 20 million generations each, sampled every 1000th generation. For the relaxed molecular clock

analyses, we estimated the coefficient of variation (CV) to inform us on the rate heterogeneity among branches (CV approaching 0 indicates that the among-branch variation in substitution rate is much smaller magnitude than the mean rate, hence, a strict clock model cannot be rejected). Parameter convergence was confirmed using TRACER 1.6 (http://tree.bio.ed.ac.uk/software/tracer/). All dating analyses were performed at the CIPRES Science Gateway computing facility[7].

Trait-dependent diversification analyses

We relied on the Binary State Speciation and Extinction (BiSSE) model[8] to estimate diversification rates associated with all three traits (i.e., ESD, epiphytism, and euglossine-bee pollination). BiSSE model built upon the premise that speciation and extinction rates depend on the state of a particular character. We tested eight models with different configurations of speciation, extinction, and transition rates between characters: (*i*) equal rates model (no effect of the trait on diversification, M_1); *(ii)* free speciation rates in lineages with and without a trait, and equal rates of extinction and transition (M_2); (*iii*) free extinction rates in lineages with and without a trait, and equal rates of speciation and transition (M_3); (*iv*) free transition rates in lineages with and without a trait, and equal rates of speciation and extinction (M_4); *(v)* free speciation and extinction rates in lineages with and without a trait, and equal rates of transition (M_5); *(vi)* free speciation and transition rates in lineages with and without a trait, and equal rates of extinction (M_6); *(vii)* free extinction and transition rates in lineages with and without a trait, and equal rates of speciation (M_7); and *(viii)* all parameters are free (M_8).

We estimated the speciation, extinction, and transition rates corresponding to the best fitting model, according to the AICc criterion. We ran Bayesian MCMC analyses on the consensus tree, using exponential priors with parameters obtained from the best fitting model, a 1000-step burn-in, and 10,000-step chain[8]. Convergence occurred within the few first steps and parameter estimates were very stable along the chain. We summarised the posterior distributions of samples to assess variation in net diversification, turnover, and transition rates across character states after the burn-in phase. We used a global sampling of 139 species (~32% of the extant diversity of Catasetinae + Cyrtopodiinae), and we accounted for missing taxa by accommodating sampling fractions for every trait as follows: (*i*) ESD: 41% of the know species with ESD sampled *vs*. 44% of the know species with alternative matting systems sampled; (*ii*) euglossine-bee pollination: 31% of the know species with this trait sampled *vs.* 33% of the know species with alternative pollination syndromes trait sampled; (*iii*) epiphytism: 40% of the know epiphyte species sampled *vs.* 45% of the known species with alternative plant habits sampled. Information on occurrence of epiphytism and euglossine-bee pollination syndrome was obtained from the literature[9,10]. We took phylogenetic and dating uncertainties into account by running these models over a series of 500 dated trees randomly selected from the BEAST dating analysis. We then selected the best-fitting model based on the corrected Akaike Informative Criterion (AICc).

Because BiSSE may be subject to Type I errors biases[11], we tested whether diversification rates associated to character states are not artefacts. To do so, we reshuffled the coding states onto the phylogeny and ran the BiSSE models. We executed 10,000 iterations, estimating the AIC of the best model at each iteration to

get the null distribution of AIC (associated with random states) and compared it with the AIC value obtained with the observed dataset.

References

1. Chang, S.-B., Chen, W.-H., Chen, H.-H., Fu, Y.-M. & Lin, Y.-S. RFLP and inheritance patterns of chloroplast DNA in intergenic hybrids of Phalaenopsis and Doritis. *Bot. Stud.* **41,** 219–223 (2000).

2. Cafasso, D., Widmer, A. & Cozzolino, S. Chloroplast DNA inheritance in the orchid Anacamptis palustris using single-seed polymerase chain reaction. *J. Hered.* **96,** 66–70 (2005).

3. Petit, R. J. *et al.* Comparative organization of chloroplast , mitochondrial and nuclear diversity in plant populations. *Mol. Ecol.* **14,** 689–701 (2005).

4. Ree, R. H. & Smith, S. A. Maximum Likelihood inference of geographic range evolution by dispersal, local extinction, and cladogenesis. *Syst. Biol.* **57,** 4–14 (2008).

5. Matzke, N. J. Model selection in historical biogeography reveals that founder-event speciation is a crucial process in island clades. *Syst. Biol.* **63,** 951–970 (2014).

6. Antonelli, A., Nylander, J. A. A., Persson, C. & Sanmartín, I. Tracing the impact of the Andean uplift on Neotropical plant evolution. *Proc. Natl. Acad. Sci. U. S. A.* **106,** 9749–9754 (2009).

7. Miller, M. A. *et al.* A RESTful API for access to phylogenetic tools via the CIPRES Science Gateway. *Evol. Bioinforma.* **11,** 43–48 (2015).

8. FitzJohn, R. G. Analysing diversification with diversitree. 1–34 (2010).

9. Pridgeon, A. M., Cribb, P. J., Chase, M. W. & Rasmussen, F. N. *Genera*

Orchidacearum: Vol. 5. Epidendroideae (part two). (Oxford University Press, 2009).

10. Ramirez, S. R. *et al.* Asynchronous diversification in a specialized plant-pollinator mutualism. *Science (80-.).* **333,** 1742–1746 (2011).

11. Maddison, W. P. & Fitzjohn, R. G. The unsolved challenge to phylogenetic correlation tests for categorical characters. *Syst. Biol.* **64,** 127–136 (2015).

12. Baldwin, B. G. Phylogenetic utility of the internal transcribed spacers of nuclear ribosomal DNA in plants: An example from the compositae. *Mol. Phylogenet. Evol.* **1,** 3–16 (1992).

13. Monteiro, S. H. N., Selbach-Schnadelbach, A., de Oliveira, R. P. & van den Berg, C. Molecular Phylogenetics of <I>Galeandra</I> (Orchidaceae: Catasetinae) based on Plastid and Nuclear DNA Sequences. *Syst. Bot.* **35,** 476–486 (2010).

14. Baldwin, B. G. & Markos, S. Phylogenetic utility of the external transcribed spacer (ETS) of 18S-26S rDNA: congruence of ETS and ITS trees of Calycadenia (Compositae). *Mol. Phylogenet. Evol.* **10,** 449–63 (1998).

15. Górniak, M., Paun, O. & Chase, M. W. Phylogenetic relationships within Orchidaceae based on a low-copy nuclear coding gene, Xdh: Congruence with organellar and nuclear ribosomal DNA results. *Mol. Phylogenet. Evol.* **56,** 784–795 (2010).

16. Neubig, K. M. *et al.* Phylogenetic utility of ycf1 in orchids: a plastid gene more variable than matK. *Plant Syst. Evol.* **277,** 75–84 (2008).

17. Hamilton, M. B. Primer Notes: Four primer pairs for the amplification of chloroplast intergenic regions with intraspecific variation. *Mol. Ecol.* **8,** 513–525 (1999).

Supplementary tables

Table S1. Species names and voucher information for material use in this study. Taxa sequenced and newly produced sequences are shown in bold face.

Species names	ITS	ETS	*Xdh*	*mat*K	*trn*L-F	*ycf*1
Acrolophia bolusii	KF318909	-	-	KF358114	-	-
Acrolophia capensis	KF318958	-	-	KF358074	-	-
Acrolophia cochlearis	KF318947	-	-	KF358059	-	-
Acrolophia lamellata	KF318964	-	-	KF358063	-	-
Acrolophia lunata	KF318965	-	-	KF358129	-	-
Acrolophia micrantha	LN831482	-	-	KF358111	-	-
Acrolophia ustulata	KF318963	-	-	KF358121	-	-
Brassia aurantiaca	AF350518	-	-	AF350597	AF350676	FJ563573
Capanemia superflua	AF350549	-	-	FJ563840	AF350707	-
Catasetum barbatum	**MF987482**	**MF987475**	-	-	-	-
Catasetum bertioguense	KU295252	KU295217	-	-	-	-
Catasetum bicolor	**MF987483**	-	**MF987494**	-	-	**MF987512**
Catasetum blackii	**KU295253**	**KU295253**	-	-	-	**KU350872**
Catasetum boyi	**MF987484**	-	**MF987495**	-	-	**MF987513**
Catasetum callosum	**KU295254**	**MF987476**	**MF987496**	-	-	**KU350873**
Catasetum cernuum	**KU295255**	**KU295219**	-	-	-	**KU350874**
Catasetum ciliatum	**KU295256**	**KU295219**	-	-	-	**KU350875**
Catasetum collare	KT768384	KT768350	KT768454	-	-	KT768491
Catasetum complanatum	**KU295257**	**KU295221**	-	-	-	**KU350876**
Catasetum denticulatum	**KU295258**	**KU295222**	**MF987497**	-	-	**KU350877**
Catasetum discolor	**KU295259**	**KU295223**	**MF987498**	-	-	**MF987514**
Catasetum expansum	**KU295260**	**KU295224**	**MF987499**	KF660300	-	**KU350878**
Catasetum fimbriatum	**KU295261**	**KU295225**	**MF987500**	-	-	**KU350879**

Catasetum galeritum	**KU295262**	**KU295226**	-	-	-	**KU350880**
Catasetum gladiatorium	**KU295263**	**KU295227**	-	-	-	**KU350881**
Catasetum globiflorum	-	-	-	-	-	**MF987515**
Catasetum gnomus	**KU295264**	**KU295228**	-	-	-	**KU350882**
Catasetum hopkinsonianum	**KU295265**	-	-	-	-	-
Catasetum integerrimum	**MF987485**	**MF987477**	-	-	-	**MF987516**
Catasetum juruenense	KT768385	KT768351	KT768455	-	-	KT768492
Catasetum laminatum	**LK054139**	-	-	-	-	-
Catasetum lanciferum	**KU295266**	**KU295229**	**MF987501**	-	-	**KU350884**
Catasetum luridum	**KU295267**	**KU295230**	-	-	-	-
Catasetum macrocarpum	KT768386	KT768352	KT768456	-	-	KT768493
Catasetum maculatum	**MF987486**	**MF987478**	**MF987502**	-	-	-
Catasetum meeae	KT768387	KT768353	KT768457	-	-	-
Catasetum ochraceum	**MF987487**	**MF987479**	**MF987503**	-	-	**MF987517**
Catasetum ornithoides	**MF987488**	-	**MF987504**	-	-	**MF987518**
Catasetum osakadianum	**KU295268**	**KU295231**	-	-	-	**KU350886**
Catasetum osculatum	**MF987489**	-	**MF987505**	-	-	**MF987519**
Catasetum pileatum	**KU295269**	-	-	-	-	**KU350887**
Catasetum purum	**KU295270**	**KU295232**	-	-	-	**KU350888**
Catasetum rodigasianum	**KU295271**	-	-	-	-	-
Catasetum rooseveltianum	**KU295272**	**KU295233**	-	-	-	**KU350890**
Catasetum x *roseoalbum*	KT768388	KT768354	KT768458	-	-	KT768494
Catasetum saccatum	**KU295273**	**KU295234**	-	-	EU441214	**KU350891**
Catasetum sanguineum	**KU295274**	-	**MF987507**	-	-	**KU350892**
Catasetum spitzii	**KU295275**	**KU295235**	**MF987508**	-	-	-
Catasetum stenoglossum	**KU295276**	**KU295235**	-	-	-	**KU350893**
Catasetum tabulare	**MF987491**	**MF987480**	**MF987509**	-	-	**MF987520**
Catasetum uncatum	**MF987492**	-	**MF987510**	-	-	-
Catasetum viridiflavum	**KU295277**	**KU295237**	-	-	-	**MF987521**

Caucaea cucullata	AF432952	-	-	AF433005	AF433024	-
Chytroglossa aurata	-	-	-	FJ564753	-	FJ563245
Chytroglossa marileoniae	DQ210301	-	-	FJ565112	FJ562408	-
Cischweinfia colombiana	FJ565464	-	-	-	-	-
Clowesia amazonica	-	-	-	-	-	**KU350895**
Clowesia dodsoniana	**MF987493**	**MF987481**	**MF987511**	KF660299	-	KF660524
Clowesia rosea	U257701	**KU257693**	-	-	-	-
Clowesia russelliana	KT768389	-	-	-	-	KT768495
Clowesia thylaciochila	LK054140	-	-	-	-	-
Comparettia langkastii	FJ565460	-	-	FJ563962	FJ562383	-
Comparettia macroplectron	AF350541	-	-	AF350620	AF350699	-
Comparettia schaeferi	FJ565337	-	-	FJ563932	FJ562368	-
Cuitlauzina candida	EF079417	-	-	EF079217	FJ562416	-
Cyanaeorchis arundinae	**KF771816**	**KF771816**	-	**KF771820**	-	**KU257725**
Cyanaeorchis minor	**KF771818**	**KF771818**	-	**KF771822**	-	**KU350898**
Cyanaeorchis praetermissa	KF771819	-	-	KF771823	-	-
Cycnoches aureum	KT768390	KT768355	KT768459	-	-	KT768496
Cycnoches barthiorum	KT768391	KT768356	KT768460	-	-	KT768497
Cycnoches chlorochilon	KT768392	KT768357	KT768461	-	-	KT768498
Cycnoches cooperi	KT768393	KT768358	KT768462	KF660301	-	KT768499
Cycnoches densiflorum	KT768394	KT768359	KT768463	-	-	-
Cycnoches dianae	KT768395	KT768360	KT768464	-	-	KT768501
Cycnoches egertonianum	KM598425	KT768360	KT768464	EU214159	EU214159	KT768501
Cycnoches guttulatum	KT768398	KT768363	KT768467	-	-	KT768504
Cycnoches haagii	KU257702	KT768364	KT768468	-	-	-
Cycnoches herrenhusanum	KU257703	KT768365	KT768469	-	-	KT768506
Cycnoches lehmannii	KT768401	KT768366	KT768470	KF660266	-	KT768507
Cycnoches loddigesii	KT768402	KT768367	KT768471	-	-	KT768508
Cycnoches manoelae	KT768403	KT768368	KT768472	KF660254	-	KT768509

Cycnoches pachydactylon	KT768404	KT768369	KT768473	KF660258	-	KT768510
Cycnoches pentadactylon	KU257704	KT768369	KT768473	-	-	KT768510
Cycnoches peruvianum	KT768405	KT768372	KT768475	-	-	KT768513
Cycnoches quatuorcristis	MF285500	MF285467	-	-	-	-
Cycnoches suarezii	KT768408	KT768374	KT768476	-	-	KT768515
Cycnoches ventricosum	KT768409	KT768375	KT768477	-	-	-
Cycnoches warszewiczii	KT768410	KT768376	KT768478	EU214322	-	KT768517
Cymbidiella pardalina	AF470489	-	-	AF470459	-	-
Cymbidium devonianum	AF470515	-	-	-	-	-
Cymbidium eburneum	AF284709	-	KT768479	-	-	-
Cymbidium tracyanum	KT768412	-	-	-	-	-
Cymbidium wenshanense	AF284708	-	-	-	-	-
Cyrtochiloides cardiochila	AF432947	-	-	AF433011	AF433030	-
Cyrtochilum aurantiacum	AF350872	-	-	AF433008	AF433027	-
Cyrtochilum cimiciferum	AF350560	-	-	AF350639	AF350718	-
Cyrtochilum edwardii	AF350557	-	-	FJ565148	AF350715	-
Cyrtochilum graminoides	AF350875	-	-	AF433013	AF433032	-
Cyrtochilum meirax	AF432959	-	-	AF433016	AF433035	-
Cyrtopodium aliciae	EU877156	-	-	-	-	**KU350900**
Cyrtopodium andersonii	AF470490	-	MF285528	KF660267	-	KF660329
Cyrtopodium glutiniferum	-	-	-	-	-	MF285554
Cyrtopodium holstii	-	-	-	-	-	**KU350901**
Cyrtopodium longibulbosum	-	-	-	KF660285	-	KF660453
Cyrtopodium macrobulbon	-	-	-	LN609766	-	-
Cyrtopodium punctatum	AF239412	-	-	AF239508	AF239604	KT750238
Cyrtopodium virescens	-	-	-	-	-	**KU350902**
Dipodium paludosum	KY988619	-	KT750227	KT750201	-	KT750239
Dressleria dilecta	AF239411	-	GU004391	EU214339	AF239603	KU257728
Dressleria fragrans	-	-	-	KF660265	-	KF660327

Dressleria helleri	-	-	-	KF660264	-	KF660326
Dressleria severiniana	MF285505	-	KU257721	-	-	KU257729
Dressleria sp	KT768413	KU25769	-	-	-	KU257730
Erycina glossomystax	FJ565586	-	-	FJ565073	FJ562398	-
Eulophia acutilabra	KF318954	-	-	LN831656	-	-
Eulophia angolensis	KF318932	KU257698	-	KF358075	-	KU257731
Eulophia calanthoides	KF318896	-	-	KF358107	-	-
Eulophia callichroma	KF318903	-	-	-	-	-
Eulophia coeloglossa	KF318961	-	-	-	-	-
Eulophia cucullata	KF318946	-	-	-	-	-
Eulophia flava	JN114508	-	-	-	-	-
Eulophia flavopurpurea	KF318930	-	-	-	-	-
Eulophia graminea	AF284727	-	-	KF358078	-	-
Eulophia guineensis	KF318960	-	-	LN831662	AF239605	EU490745
Eulophia hereroensis	KF318940	-	-	KF358066	-	-
Eulophia hians	KF318892	-	-	KF358060	-	-
Eulophia horsfallii	KF318922	-	-	LN831636	-	-
Eulophia livingstoneana	LN831402	-	-	LN849252	-	-
Eulophia longisepala	KF318918	-	-	KF358086	-	-
Eulophia macowanii	KF318904	-	-	KF358120	-	-
Eulophia meleagris	KF318936	-	-	KF358083	-	-
Eulophia ovalis	KF318905	-	-	KF358064	-	-
Eulophia parviflora	KF318893	-	-	KF358062	-	-
Eulophia parvilabris	KF318927	-	-	KF358130	-	-
Eulophia ruwenzoriensis	EU877159	-	-	-	-	-
Eulophia schnelliae	KF318891	-	-	KF358126	-	-
Eulophia schweinfurthii	KF318924	-	-	KF358133	-	-
Eulophia speciosa	KF318967	-	-	KF358123	-	-
Eulophia stachyodes	KF318953	-	-	-	-	-

Eulophia streptopetala	KF318934	-	-	EF079258	-	**MF987522**
Eulophia tenella	KF318900	-	-	KF358119	-	-
Eulophia tuberculata	KF318945	-	-	KF358076	-	-
Eulophia zeyheriana	-	-	-	KF358091	-	-
Fernandezia ecuadorensis	FJ565635	-	-	FJ565127	FJ562413	-
Fernandezia ionanthera	FJ565236	-	-	AF239486	AF239582	-
Fernandezia tica	FJ565453	-	-	FJ564944	DQ315917	-
Galeandra batemanii	-	EU877122	-	-	-	-
Galeandra baueri	EU877138	EU877121	-	-	-	-
Galeandra beyrichii	EU877151	EU877133	-	-	-	-
Galeandra blanchetii	EU877140	EU877123	-	-	-	-
Galeandra cristata	-	EU877124	-	-	-	-
Galeandra devoniana	EU877142	KT768378	-	EU877181	-	KF660330
Galeandra greenwoodiana	EU877144	EU877127	-	-	-	-
Galeandra junceaoides	EU877145	EU877128	-	-	-	-
Galeandra leptoceras	-	KU257699	KU257722	-	-	KX495755
Galeandra macroplectra	-	-	-	-	-	KX495756
Galeandra magnicolumna	EU877146	EU877129	-	-	-	-
Galeandra minax	EU877147	EU877130	-	-	-	**KU350903**
Galeandra paraguayensis	EU877153	EU877134	-	-	-	-
Galeandra santarena	EU877143	EU877126	-	-	-	-
Galeandra stangeana	EU877148	EU877132	-	-	-	-
Galeandra styllomisantha	EU877154	-	-	-	-	-
Geodorum densiflorum	LN831388	-	-	JN004440	-	-
Geodorum recurvum	KF560539	-	-	KF673833	KF650564	-
Gomesa dasytyle	AF350551	-	-	AF350630	AF350709	-
Gomesa radicans	FJ565624	-	-	FR832799	FJ562410	-
Grammatophyllum papuanum	-	-	-	EF079262	-	-
Grammatophyllum speciosum	AF470488	-	-	AF470458	-	-

Grandiphyllum auricula	FJ565663	-	-	FJ565155	FJ562382	-
Grandiphyllum divaricatum	FJ565455	-	-	FJ564946	FJ562381	-
Grobya amherstiae	EU877158	EU877137	-	-	-	-
Grobya cipoensis	-	-	KU295244	-	-	-
Grobya fascifera	-	-	KU295245	-	-	-
Grobya galeata	AF470487	-	KU295246	AF470457	-	-
Hofmeisterella eumicroscopica	AF350510	-	-	FJ565091	AF350668	-
Ionopsis minutiflora	AF350548	-	-	AF350627	AF350706	-
Lockhartia micrantha	FJ565172	-	-	FJ564691	FJ562347	-
Macradenia rubescens	FJ565345	-	-	FJ564839	FJ562370	-
Macroclinium aurorae	FJ565626	-	-	FJ565118	-	-
Macroclinium dalstroemii	FJ565585	-	-	FJ565072	-	-
Miltonia phymatochila	FJ565222	-	-	FJ563856	FJ562409	-
Miltoniopsis roezlii	FJ565671	-	-	FJ565165	FJ562417	FJ563833
Mormodes andreettae	LK054149	-	-	-	LK054186	-
Mormodes aromatica	KM598430	-	-	AY368417	LK054209	-
Mormodes badia	KT768415	KT768380	KT768484	-	LK054181	KT768525
Mormodes buccinator	LK054148	LK054148	-	-	-	**KU350904**
Mormodes castroi	LK054150	-	-	-	LK054187	-
Mormodes colossus	LK054157	-	-	-	LK054194	-
Mormodes dasilvae	LK054151	**LK054151**	-	-	LK054188	**KU350905**
Mormodes elegans	LK054152	**LK054152**	-	-	LK054189	-
Mormodes escobarii	LK054159	-	-	-	LK054196	-
Mormodes fractiflexa	LK054160	-	-	-	LK054197	-
Mormodes hookeri	LK054161	-	-	-	LK054198	-
Mormodes horichii	LK054162	-	-	-	LK054199	-
Mormodes ignea	LK054163	-	-	-	LK054200	-
Mormodes lawrenceana	LK054146	-	-	-	-	-
Mormodes lineata	LK054164	-	-	-	-	-

Mormodes lobulata	LK054165	-	-	-	LK054201	-
Mormodes luxata	KT768417	KT768382	KT768486	-	-	KT768527
Mormodes maculata	LK054170	-	-	-	LK054206	-
Mormodes nagelii	LK054172	-	-	-	LK054208	-
Mormodes oestlundiana	LK054144	-	-	-	LK054182	-
Mormodes paraensis	LK054153	LK054153	-	-	**LK054190**	-
Mormodes pardalinata	LK054175	-	-	-	LK054210	-
Mormodes punctata	KT768418	KT768383	KT750231	-	-	-
Mormodes rolfeana	LK054156	-	-	-	-	-
Mormodes sanguineoclaustra	LK054176	-	-	-	LK054211	-
Mormodes skinneri	LK054167	-	-	-	LK054203	-
Mormodes sotoana	LK054168	-	-	-	LK054204	-
Mormodes tapoayensis	LK054154	-	-	-	LK054191	**KU350906**
Mormodes tezontle	LK054145	-	-	-	LK054183	-
Mormodes theiochlora	LK054147	-	-	-	LK054185	-
Mormodes tuxtlensis	LK054171	-	-	-	LK054207	-
Mormodes uncia	LK054177	-	-	-	LK054212	-
Mormodes variabilis	LK054169	-	-	-	-	-
Mormodes warszewiczii	LK054155	-	-	-	LK054192	-
Mormodes williamsii	LK054178	-	-	-	LK054213	-
Nohawilliamsia orthostates	FJ565399	-	-	FJ563950	FJ562375	-
Notylia barkeri	AF350545	-	-	AF350624	FJ564794	-
Notylia ecuadorensis	FJ565477	-	-	FJ564961	FJ562385	-
Notyliopsis beatricis	FJ565597	-	-	FJ565086	FJ562401	-
Oeceoclades bernetii	KF318937	-	-	KF358068	-	-
Oeceoclades maculata	KF318917	-	-	-	-	-
Oeceoclades pulchra	KF318911	-	-	KF358079	-	KT768523
Oliveriana brevilabia	FJ565321	-	-	EF079202	FJ562367	-
Oncidium chrysomorphum	AF350796	-	-	FJ564996	AF433039	-

Oncidium epidendroides	AF350777	-	-	FJ564802	FJ562387	-
Oncidium harryanum	EF079421	-	-	EF079216	AF350727	-
Oncidium lehmannianum	FJ565395	-	-	FJ564891	FJ562373	-
Oncidium multistellare	AF350729	-	-	EF079213	AF350729	-
Oncidium noezlianum	AF350728	-	-	EF079211	AF350728	-
Oncidium peruvianoides	FJ565512	-	-	FJ564995	FJ562392	-
Oncidium sphacelatum	AF350564	-	-	AF350643	AF350722	-
Oncidium strictum	AF350568	-	-	FJ564985	AF350726	-
Ornithocephalus bicornis	FJ565564	-	-	FJ565049	FJ562397	-
Ornithocephalus suarezii	FJ565560	-	-	FJ565048	FJ562396	-
Orthochilus aculeatus	KF318949	-	-	-	-	-
Orthochilus chloranthus	KF318939	-	-	-	-	-
Orthochilus ensatus	KF318956	-	-	-	-	-
Orthochilus foliosus	KF318920	-	-	KF358101	-	-
Orthochilus leontoglossus	KF318941	-	-	KF358132	-	-
Orthochilus mechowii	KF318914	-	-	-	-	-
Orthochilus odontoglossus	KF318951	-	-	-	-	-
Orthochilus tabularis	KF318907	-	-	-	-	-
Otoglossum chiriquense	AF350561	-	-	AF433017	AF350719	-
Otoglossum coronarium	EF079410	-	-	FJ565100	FJ562406	-
Otoglossum harlingii	AF350879	-	-	-	-	-
Phymatidium falcifolium	FJ565362	-	-	-	-	-
Plectrophora cultrifolia	FJ565495	-	-	-	-	-
Plectrophora triquetra	FJ565494	-	-	-	-	-
Polyotidium huebneri	FJ565458	-	-	FJ565458	-	-
Polystachya cultriformis	GU556643	-	-	-	-	KF660306
Psychopsiella limminghei	FJ565660	-	-	FJ565152	-	FJ563600
Psychopsis papilio	AF350582	-	-	-	AF350740	-
Psychopsis sanderae	FJ565198	-	-	-	-	-

Pterostemma antioquiense	FJ565396	-	-	FJ563948	FJ562374	-
Rhynchostele londesboroughiana	AF350530	-	-	EF065561	AF350688	-
Rodriguezia arevaloi	FJ565331	-	-	FJ564824	-	-
Rodriguezia delcastilloi	AF350543	-	-	AF350622	AF350701	-
Rodriguezia satipoana	AF350544	-	-	AF350544	AF350702	-
Solenidium lunatum	FJ565359	-	-	FJ563940	-	-
Solenidium portillae	FJ565472	-	-	FJ564956	FJ562384	-
Systeloglossum acuminatum	AF350528	-	-	AF350607	AF350686	-
Systeloglossum bennettii	FJ565214	-	-	FJ564728	-	-
Telipogon bowmanii	-	-	-	DQ315912	DQ315938	-
Telipogon hystrix	DQ315841	-	-	DQ315899	DQ315925	-
Telipogon nervosus	DQ315870	-	-	HQ219263	DQ315933	-
Telipogon obovatus	FJ565603	-	-	FJ565093	-	-
Telipogon pogonostalix	AF350511	-	-	AF239488	AF350669	-
Telipogon pulcher	DQ315875	-	-	DQ315910	DQ315936	-
Telipogon venustus	FJ565183	-	-	FJ564703	-	-
Thysanoglossa jordanensis	EF079389	-	-	EF079224	-	-
Tolumnia variegata	AF350533	-	-	AF350612	AF350691	-
Trichoceros muralis	FJ565468	-	-	FJ563966	DQ315939	FJ562901
Trichopilia brevis	AF350581	-	-	EF079229	AF350739	-
Trichopilia fragrans	FJ565568	-	-	FJ565053	-	-
Trichopilia sanguinolenta	AF350580	-	-	-	-	-
Vitekorchis lucasiana	FJ565573	-	-	-	-	-
Warmingia eugenii	FJ565196	-	-	FJ563841	-	-
Zygostates alleniana	EF079390	-	-	EF079225	DQ315941	-
Zygostates grandiflora	AF350508	-	-	AF350587	AF350666	-

Table S2. Primer and PCR settings used for amplifying plastid and nuclear DNA loci.

Loci	Primer	Sequence	Reference	Pre-melt	Amplification	Final extension	Number of amplification cycles
ITS	ITS 4	TCC-TCC-GCT-TAT-TGA-TAT-GC	Baldwin, 1992[12]	95°C (3 min)	95°C (30 secs) + 52°C (1 min) + 68°C (1 min)	68°C (10 min)	39
	ITS 5	GGA-AGT-AAA-AGT-CGT-AAC-AAG-G		95°C (3 min)	95°C (30 secs) + 52°C (1 min) + 68°C (1 min)	68°C (10 min)	39
ETS	EST-Orchid	CAT-ATG-AGT-TGT-TGC-GGA-CC (AT)-T	Monteiro et al. 2010[13]	95°C (3 min)	95°C (30 secs) + 52°C (1 min) + 68°C (1 min)	68°C (10 min)	39
	18-IGS	AGA-CAA-GCA-TAT-GAC-TAC-TGG-CAG-G	Baldwin and Markos, 1998[14]	95°C (3 min)	95°C (30 secs) + 52°C (1 min) + 68°C (1 min)	68°C (10 min)	39
Xdh	X502F	TGT-GAT-GTC-GAT-GTA-TGC	Górniak et al. 2010[15]	95°C (3 min)	95°C (30 secs) + 53°C (1 min) + 68°C (1.5 min)	68°C (10 min)	39
	X1599R	G(AT)G-AGA-GAA-A(CT)TG-GAG-CAA-C		95°C (3 min)	95°C (30 secs) + 53°C (1 min) + 68°C (1.5 min)	68°C (10 min)	39
*ycf*1	3720F	TAC-GTA-TGT-AAT-GAA-CGA-ATG-G	Neubig et al. 2009[16]	95°C (3 min)	95°C (30 secs) + 54°C (1 min) + 68°C (1.5 min)	68°C (10 min)	39
	5500R	GCT-GTT-ATT-GGC-ATC-AAA-CCA-ATA-GCG		95°C (3 min)	95°C (30 secs) + 54°C (1 min) + 68°C (1.5 min)	68°C (10 min)	39
*trn*S-G	trn-S(GCU)	GCC-GCT-TTA-GTC-CAC-TCA-GC	Hamilton, 1999[17]	95°C (3 min)	95°C (30 secs) + 51.5°C (1 min) + 68°C (1.5 min)	68°C (10 min)	39
	trn-G(UCC)	GAA-CGA-ATC-ACA-CTT-TTA-CCA-C		95°C (3 min)	95°C (30 secs) + 51.5°C (1 min) + 68°C (1.5 min)	68°C (10 min)	39

Table S3. Alignment characterisation.

**Partition**	**Model**	**Likelihood**
ITS	GTR+G	-22881.15
ETS	TIM3+G	-4003.25
Xdh	TPM2uf+G	-3555.32
*mat*K	TVM+G	-14706.06
*trn*L-F	TPM1uf+G	-10074.65
*ycf*1	TPM1uf+G	-7596.36

Table S4. Likelihood of biogeographical models (DEC, DIVA, BAYAREA) as implemented in BIOGEOBEARS.

Model H_1	Model H_0	logL[1] H_1	logL H_0	D-statistic	*p*-value	AIC H_1	AIC H_0
DEC+J	DEC	-875.5	-893.9	36.78	1.3e-09	1757	1792
DIVALIKE+J	DIVALIKE	-912.3	-926.6	28.62	8.8e-08	1831	1857
BAYAREALIKE+J	BAYAREALIKE	*-860.3*	-931	141.5	1.3e-32	*1727*	1866

[1]Log Likelihood

Table S5. Model selection and parameter values of BiSSE analysis on lineages with ESD and alternative sexual systems.

Model	logL[1]	AICc[2]	λ^3 (0)	λ (1)	μ^4 (0)	μ (1)	$q_{0\text{-}1}$[5]	$q_{1\text{-}0}$[6]
M_1	-262.414	531.022	-	2.2724	-	2.1596	-	0.01347
M_2	**-248.854**	**506.035**	1.2539	2.0244	-	1.1816	-	0.00586
M_3	-250.101	508.528	-	1.5893	1.5308	0.6345	-	0.00366
M_4	-260.683	529.691	-	2.2688	-	2.1554	0.02002	0.000001925
M_5	-248.654	507.800	1.2327	2.0959	1.1590	1.2670	-	1.26707
M_6	-248.273	507.039	1.2496	2.0113	-	1.1742	0.00689	0.000002158
M_7	-249.814	510.120	-	1.5992	1.5393	0.6592	0.00403	0.000006212
M_8	-247.921	508.538	1.1827	2.2634	1.1007	1.4974	0.00904	0.000005874

[1]log Likelihood

[2]Akaike Informative Criterion corrected

[3]Speciation rates

[4]Extinction rates

[5]transition rates from state 0 to 1

[6]transition rates from state 1 to 0

Table S6. Model selection and parameter values of BiSSE analysis on lineages pollinated by male euglossine bees and alternative pollination syndromes.

Model	logL[1]	AICc[2]	λ^3 (0)	λ (1)	μ^4 (0)	μ (1)	q_{0-1}[5]	q_{1-0}[6]
M_1	-245.514	497.222	-	2.2924	-	2.1767	-	0.0030665
$\boldsymbol{M_2}$	**-240.045**	**488.416**	1.8276	2.1389	-	1.8172	-	0.0020853
M_3	-240.741	489.807	-	2.0960	2.0865	1.7851	-	0.0019566
M_4	-244.693	497.712	-	2.2919	-	2.1762	0.006970	0.000002017
M_5	-238.972	488.437	1.2969	2.4619	1.2625	2.1751	-	2.1751137
M_6	-239.715	489.922	1.8364	2.1388	-	1.8221	0.003017	0.000003762
M_7	-240.449	491.390	-	2.1084	2.0950	1.8048	0.002745	0.0000034742
M_8	-238.273	489.241	1.2101	2.5350	1.1618	2.2638	0.006002	0.0000026449

[1]log Likelihood

[2]Akaike Informative Criterion corrected

[3]Speciation rates

[4]Extinction rates

[5]transition rates from state 0 to 1

[6]transition rates from state 1 to 0

Table S7. Model selection and parameter values of BiSSE analysis on epiphytic lineages and alternative plant habits.

Model	logL[1]	AICc[2]	λ^3 (0)	λ (1)	μ^4 (0)	μ (1)	$q_{0\text{-}1}$[5]	$q_{1\text{-}0}$[6]
M_1	-258.626	523.446	-	1.7808	-	1.6653	-	0.01611
M_2	-256.090	520.505	1.2590	1.5762	-	1.2320	-	0.01462
M_3	-256.221	520.768	-	1.5223	1.5019	1.1720	-	0.01326
M_4	-257.123	522.572	-	1.7813	-	1.6656	0.04960	0.000579
M_5	-255.714	521.919	1.1414	1.6908	1.1089	1.4030	-	1.40300
$\boldsymbol{M_6}$	**-253.720**	**517.931**	1.2762	1.5697	-	1.2374	0.02558	0.00001974
M_7	-254.303	519.098	-	1.5598	1.5259	1.2443	0.02234	0.00002881
M_8	-253.018	518.731	0.8744	1.7400	0.8010	1.4450	0.043234	0.00016626

[1]log Likelihood

[2]Akaike Informative Criterion corrected

[3]Speciation rates

[4]Extinction rates

[5]transition rates from state 0 to 1

[6]transition rates from state 1 to 0

Supplementary figures

Fig. S1. Conflicting nuclear-plastid associations (i.e. pair of nuclear / plastid taxa) obtained by PACo analysis, using Bayesian posterior probability trees. Taxa with normalised squared residual scores above the threshold value (red line) indicate potential conflicting associations.

Fig. S2. Phylogenetic relationships of Catasetinae and sister subtribes (Eulophiinae, Dipodiinae, Oncidiinae, Cyrtopodiinae, Cymbidiinae) independently derived from nuclear and plastid datasets. potential outlier, congruent and excluded terminals (i.e taxa with sequences included on the plastid loci but missing on the nuclear dataset prior analysis) are shown in red, green and blue, respectively.

Fig. S3. Best scoring ML tree of Catasetinae and sister subtribes (Eulophiinae, Dipodiinae, Oncidiinae, Cyrtopodiinae, Cymbidiinae) obtained from non-conflicting concatenated nuclear and plastid loci. Node charts indicate Bootstrap Support (BS > 75), where fully black diagrams indicate BS of 100.

Fig. S4. Chronogram of Catasetinae and sister subtribes obtained under a relaxed clock model, applied to a non-conflicting, concatenated nuclear and plastid loci. Minimum and maximum age intervals are provided. MRCA of Catasetinae as well as multiple origins of ESD are shown.

Fig. S5. Probabilities of areas estimated as inferred under the BayArea+J model using a relaxed clock model dated phylogeny. Grey colours at pie diagrams indicate probabilities of shared ancestral areas (i.e. two or more biogeographical regions) estimated at nodes. The LCA of Catasetinae is indicated with a black star and gains of ESD are indicated with red circles. Coded biogeographical areas are colour-coded following inset map, and are shown in front of taxa names (grey colours indicate taxa

distributed in more than one biogeographical region). Sub-tribe members sampled in our phylogeny are color-coded. Right panels show selected representatives of (a) Cymbidiinae (*Grammatophyllum measuresianum*); (b) Cyrtopodiinae (*Cyrtopodium polyphyllum*); (c) Eulophiinae (*Eulophia streptopetala*); (d, e, f) Catasetinae (*Catasetum fimbriatum*, *Galeandra macroplectron*, and *Mormodes punctata,* respectively); (h, i) Oncidiinae (*Trichoceros* sp., *Rossioglossum grande*). Photos (except b by L. Varella): O. Pérez. [Inset: Codes areas used for biogeographical analysis. Geopolitical boundaries map generated by ArcMAP (http://www.sri.com) using political divisions and elevation data from DIVA-GIS (http:www.diva-gis.org/data)].

Fig. S6. Maximum Likelihood optimisation (1000 iterations) under an Asymmetrical Rate Model (ARD) of Environmental Sex Determination (ESD) of Catasetinae on a BEAST maximum credibility clade chronogram. Posterior probabilities of each state are show at nodes. The LCA of Catasetinae is indicated with a black star.

Fig. S7. Bioclimatic variables and their corresponding contributions to Axis 1 (47.9% variance) of PCA analysis. Note that there is no evident discrimination between temperature and seasonality.

Fig. S8. Bioclimatic variables and their corresponding contributions to Axis 2 (23.6% variance) of PCA analysis. Note that there is no evident discrimination between temperature and seasonality.

Fig. S9. Presence and absence of Environmental Sex Determination overlaid on: (a) A Principal Component Analysis (PCA), and (b) a non-dimensional metric scaling analyses (NMDS) plot. Note that in both analyses, climatic niche spaces of lineages

with and without ESD overlap (normal confidence ellipses drawn at a confidence level of 0.95 largely intersect).

Fig. S10. Mean ancestral values and 95% confidence intervals (CI) of bioclimatic variables estimated at all nodes of the Catasetinae phylogeny by ACE analyses. (A) Altitude; (B) Annual mean temperature (Bio 1); (C) Mean diurnal range (Bio 2); (D) Isothermality (Bio 3); (E) Temperature seasonality (Bio 4); (F) Maximum temperature of warmest month (Bio 5); (G) Maximum temperature of coldest month (Bio 6); (H) Temperature annual range (Bio 7); (I) Mean temperature of wettest quarter (Bio 8); (J) Mean temperature of driest quarter (Bio 9); (K) Mean temperature of warmest quarter (Bio 10); (L) Mean temperature of coldest quarter (Bio 11); (M) Annual precipitation (Bio 12); (N) Precipitation of wettest month (Bio 13); (O) Precipitation of driest month (Bio 14); (P) Precipitation seasonality (Bio 15); (Q) Precipitation of wettest quarter (Bio 16); (R) Precipitation of driest quarter (Bio 17); (S) Precipitation of warmest quarter (Bio 18); (T) Precipitation of coldest quarter (Bio 19). Mean ancestral values are indicated by red bars. Blue circles represent mean ancestral values at ancestors where ESD originated independently. Blue bars indicate CI of ancestral values at nodes with ESD as inferred by character state estimation analyses, and orange bars indicate CI of mean ancestral values at nodes without ESD. Note the little differences between ancestral mean values of bioclimatic variables in nodes inferred to have ESD, and those inferred to have alternative sexual systems (i.e. absence of ESD).

Fig. S11. Density probability plots of speciation rates (A); differences in speciation and extinction rates (B); net diversification rates (C), and differences in net diversification rates (D) in lineages with Environmental Sex Determination and alternative sexual systems as inferred by BiSSE.

Fig. S12. Density probability plots of speciation rates (A); differences in speciation and extinction rates (B); net diversification rates (C), and differences in net diversification rates (D) in euglossine-bee pollinated lineages and alternative pollination syndromes as inferred by BiSSE.

Fig. S13. Density probability plots of speciation rates (A); differences in speciation and extinction rates (B); net diversification rates (C), and differences in net diversification rates (D) in epiphytic lineages and those with other habits as inferred by BiSSE.

Fig. S14. Null distribution of AIC of BiSSE simulations in A) ESD, and B) euglossine-bee pollination syndrome.

Figure S1

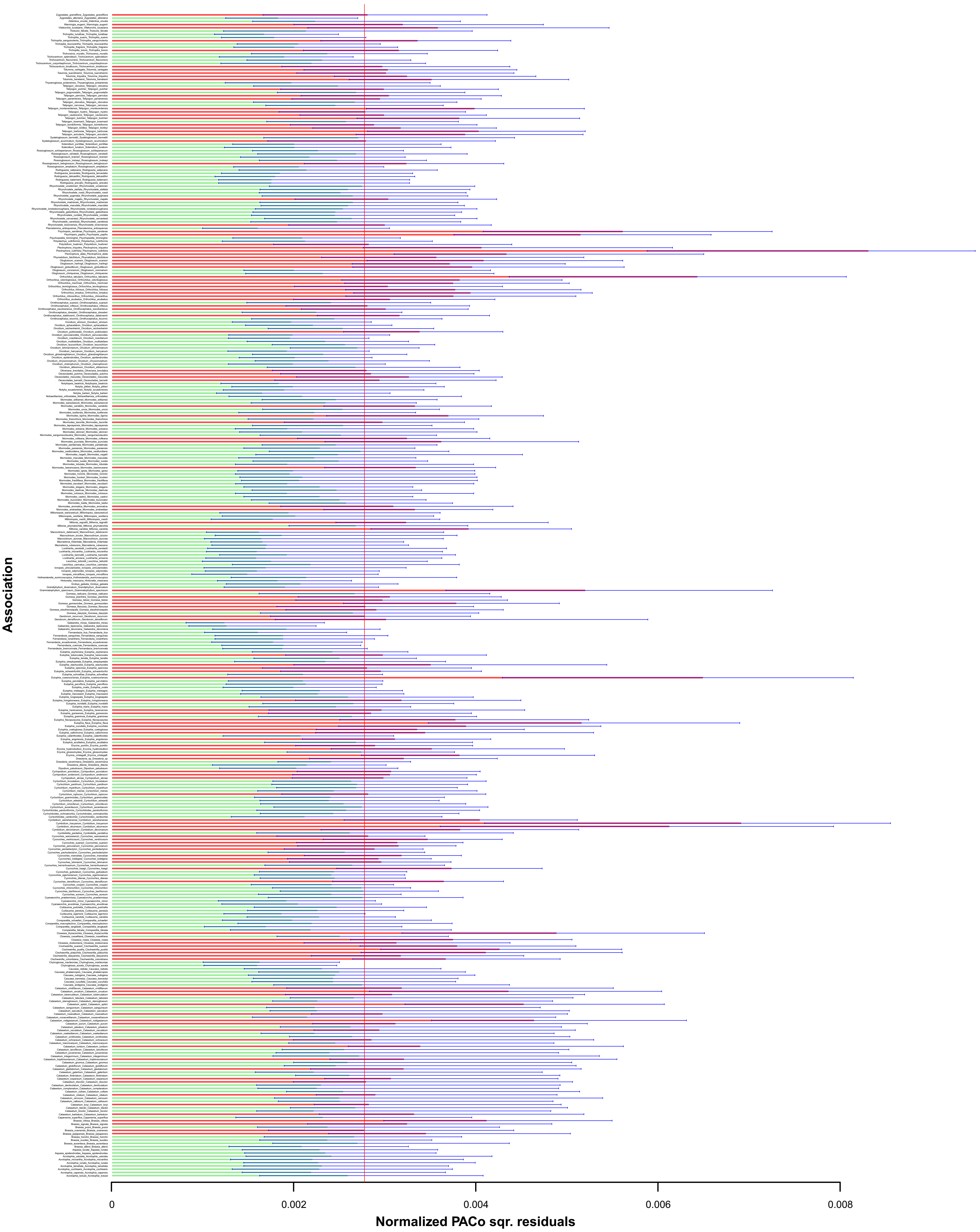
Association
0
0.002
0.004
0.006
0.008
Normalized PACo sqr. residuals

Figure S2

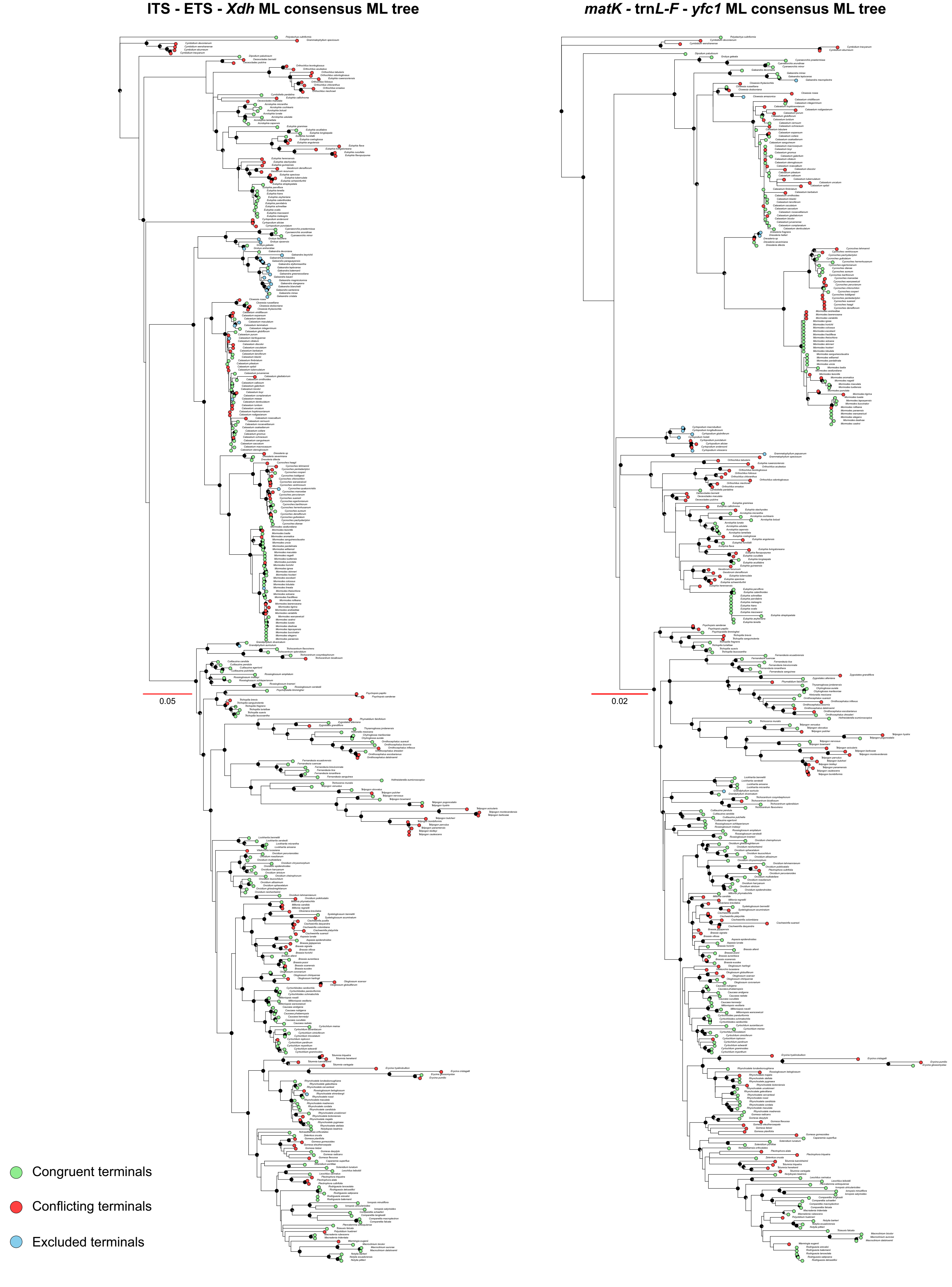
ITS - ETS - Xdh ML consensus ML tree
matK - trnL-F - yfc1 ML consensus ML tree
0.05
0.02
Congruent terminals
Conflicting terminals
Excluded terminals

Figure S3

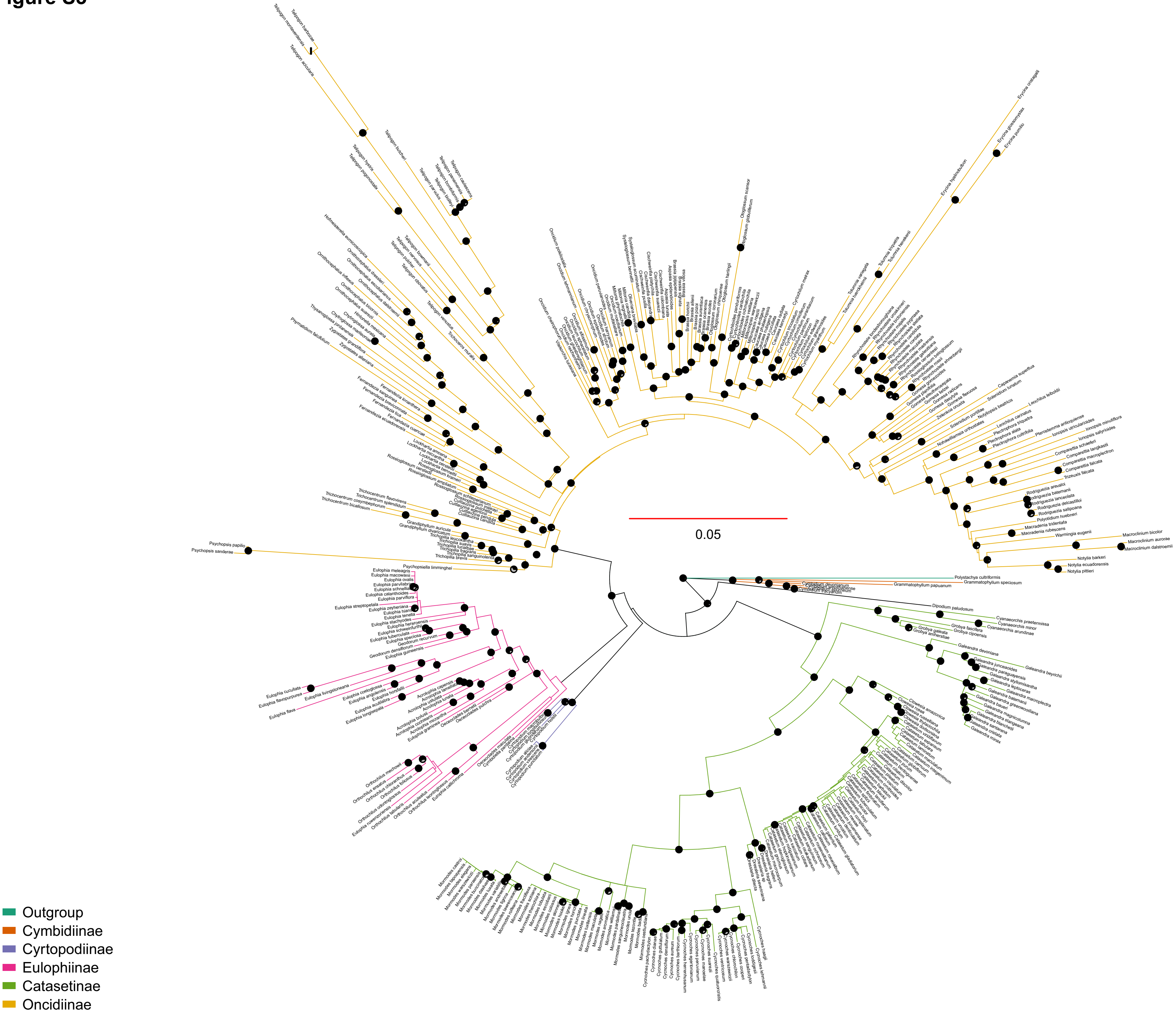
0.05
Outgroup
Cymbidiinae
Cyrtopodiinae
Eulophiinae
Catasetinae
Oncidiinae

Figure S4

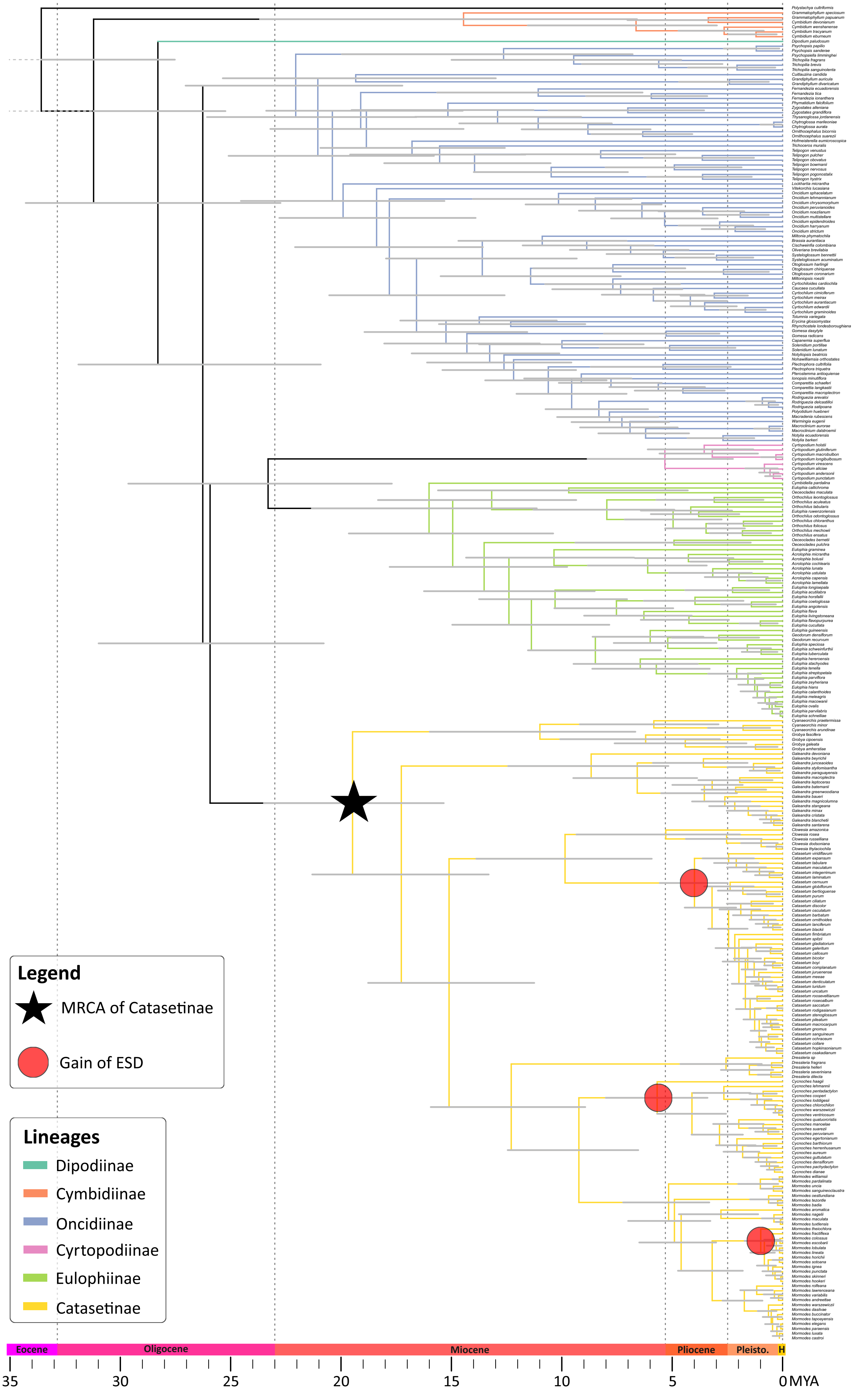
Legend
MRCA of Catasetinae
Gain of ESD
Lineages
Dipodiinae
Cymbidiinae
Oncidiinae
Cyrtopodiinae
Eulophiinae
Catasetinae
Eocene
Oligocene
Miocene
Pliocene
Pleisto.
H
35
30
25
20
15
10
5
0
MYA

Figure S5

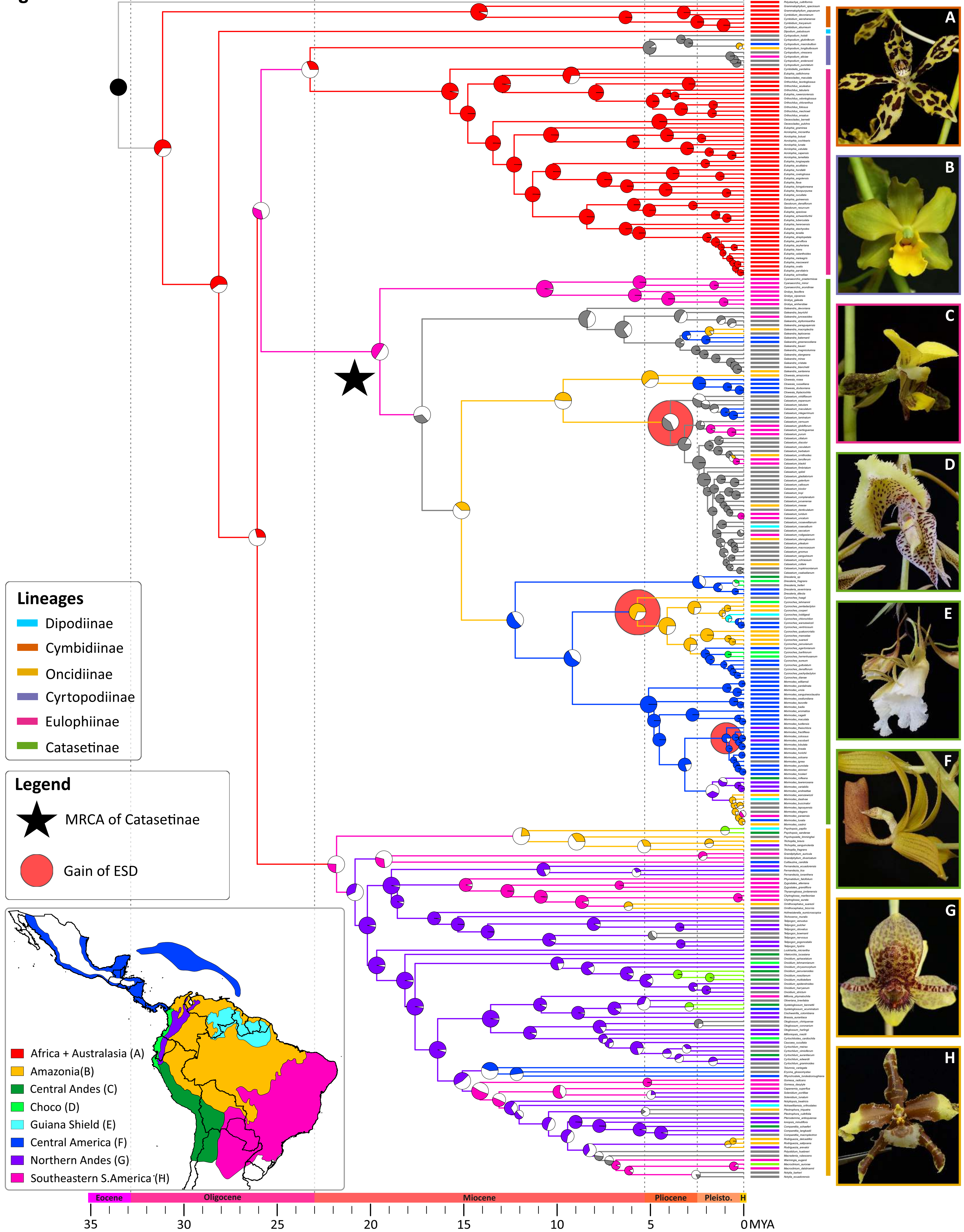
Lineages
Dipodiinae
Cymbidiinae
Oncidiinae
Cyrtopodiinae
Eulophiinae
Catasetinae
Legend
MRCA of Catasetinae
Gain of ESD
Africa + Australasia (A)
Amazonia(B)
Central Andes (C)
Choco (D)
Guiana Shield (E)
Central America (F)
Northern Andes (G)
Southeastern S.America (H)
A
B
C
D
E
F
G
H
Eocene
Oligocene
Miocene
Pliocene
Pleisto.
H
35
30
25
20
15
10
5
0 MYA

Figure S6

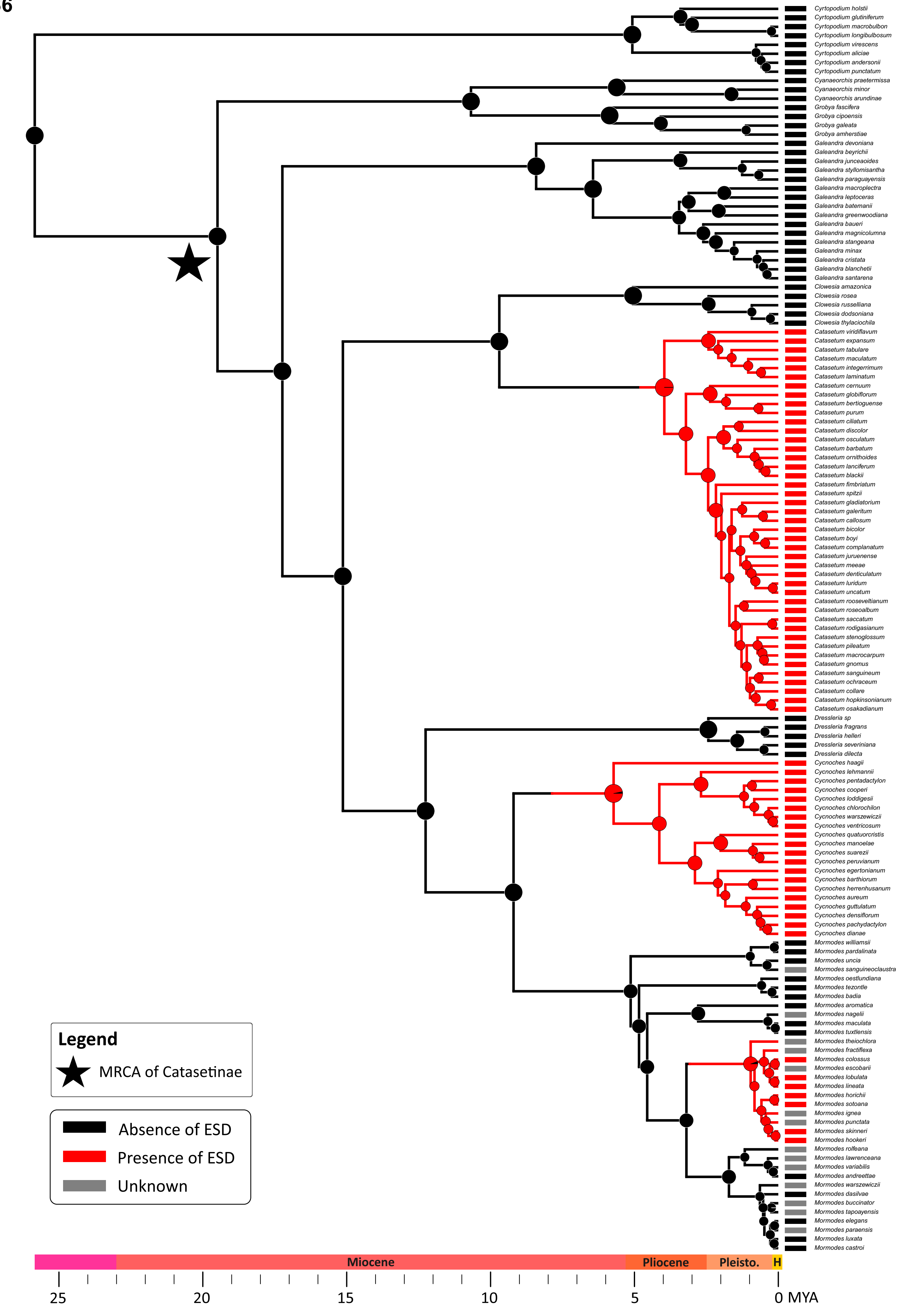

Cyrtopodium holstii
Cyrtopodium glutiniferum
Cyrtopodium macrobulbon
Cyrtopodium longibulbosum
Cyrtopodium virescens
Cyrtopodium aliciae
Cyrtopodium andersonii
Cyrtopodium punctatum
Cyanaeorchis praetermissa
Cyanaeorchis minor
Cyanaeorchis arundinae
Grobya fascifera
Grobya cipoensis
Grobya galeata
Grobya amherstiae
Galeandra devoniana
Galeandra beyrichii
Galeandra junceaoides
Galeandra styllomisantha
Galeandra paraguayensis
Galeandra macroplectra
Galeandra leptoceras
Galeandra batemanii
Galeandra greenwoodiana
Galeandra baueri
Galeandra magnicolumna
Galeandra stangeana
Galeandra minax
Galeandra cristata
Galeandra blanchetii
Galeandra santarena
Clowesia amazonica
Clowesia rosea
Clowesia russelliana
Clowesia dodsoniana
Clowesia thylaciochila
Catasetum viridiflavum
Catasetum expansum
Catasetum tabulare
Catasetum maculatum
Catasetum integerrimum
Catasetum laminatum
Catasetum cernuum
Catasetum globiflorum
Catasetum bertioguense
Catasetum purum
Catasetum ciliatum
Catasetum discolor
Catasetum osculatum
Catasetum barbatum
Catasetum ornithoides
Catasetum lanciferum
Catasetum blackii
Catasetum fimbriatum
Catasetum spitzii
Catasetum gladiatorium
Catasetum galeritum
Catasetum callosum
Catasetum bicolor
Catasetum boyi
Catasetum complanatum
Catasetum juruenense
Catasetum meeae
Catasetum denticulatum
Catasetum luridum
Catasetum uncatum
Catasetum rooseveltianum
Catasetum roseoalbum
Catasetum saccatum
Catasetum rodigasianum
Catasetum stenoglossum
Catasetum pileatum
Catasetum macrocarpum
Catasetum gnomus
Catasetum sanguineum
Catasetum ochraceum
Catasetum collare
Catasetum hopkinsonianum
Catasetum osakadianum
Dressleria sp
Dressleria fragrans
Dressleria helleri
Dressleria severiniana
Dressleria dilecta
Cycnoches haagii
Cycnoches lehmannii
Cycnoches pentadactylon
Cycnoches cooperi
Cycnoches loddigesii
Cycnoches chlorochilon
Cycnoches warszewiczii
Cycnoches ventricosum
Cycnoches quatuorcristis
Cycnoches manoelae
Cycnoches suarezii
Cycnoches peruvianum
Cycnoches egertonianum
Cycnoches barthiorum
Cycnoches herrenhusanum
Cycnoches aureum
Cycnoches guttulatum
Cycnoches densiflorum
Cycnoches pachydactylon
Cycnoches dianae
Mormodes williamsii
Mormodes pardalinata
Mormodes uncia
Mormodes sanguineoclaustra
Mormodes oestlundiana
Mormodes tezontle
Mormodes badia
Mormodes aromatica
Mormodes nagelii
Mormodes maculata
Mormodes tuxtlensis
Mormodes theiochlora
Mormodes fractiflexa
Mormodes colossus
Mormodes escobarii
Mormodes lobulata
Mormodes lineata
Mormodes horichii
Mormodes sotoana
Mormodes ignea
Mormodes punctata
Mormodes skinneri
Mormodes hookeri
Mormodes rolfeana
Mormodes lawrenceana
Mormodes variabilis
Mormodes andreettae
Mormodes warszewiczii
Mormodes dasilvae
Mormodes buccinator
Mormodes tapoayensis
Mormodes elegans
Mormodes paraensis
Mormodes luxata
Mormodes castroi
Legend
MRCA of Catasetinae
Absence of ESD
Presence of ESD
Unknown
Miocene
Pliocene
Pleisto.
H
25
20
15
10
5
0 MYA

Figure S7

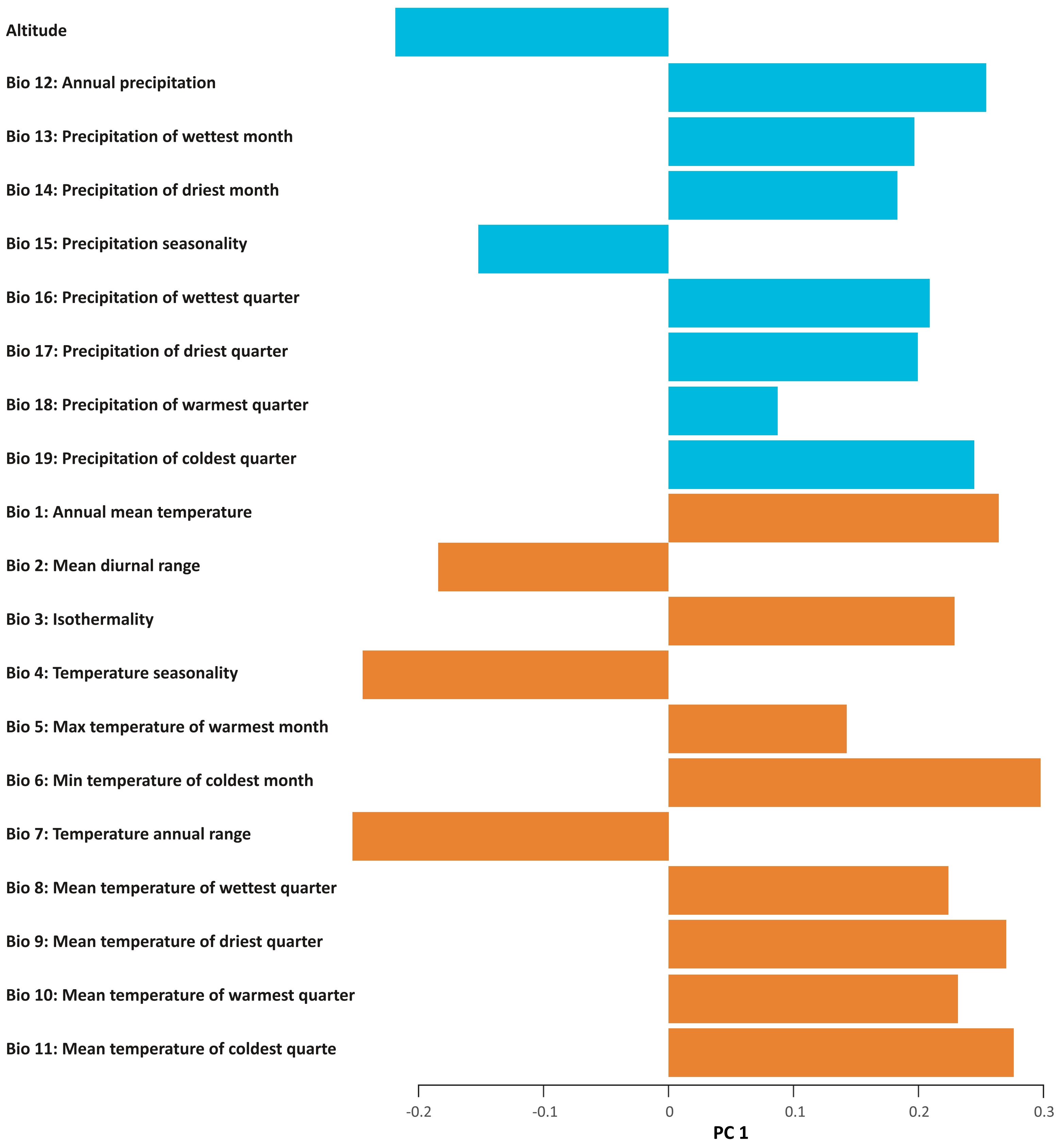
Altitude
Bio 12: Annual precipitation
Bio 13: Precipitation of wettest month
Bio 14: Precipitation of driest month
Bio 15: Precipitation seasonality
Bio 16: Precipitation of wettest quarter
Bio 17: Precipitation of driest quarter
Bio 18: Precipitation of warmest quarter
Bio 19: Precipitation of coldest quarter
Bio 1: Annual mean temperature
Bio 2: Mean diurnal range
Bio 3: Isothermality
Bio 4: Temperature seasonality
Bio 5: Max temperature of warmest month
Bio 6: Min temperature of coldest month
Bio 7: Temperature annual range
Bio 8: Mean temperature of wettest quarter
Bio 9: Mean temperature of driest quarter
Bio 10: Mean temperature of warmest quarter
Bio 11: Mean temperature of coldest quarte
-0.2
-0.1
0
0.1
0.2
0.3
PC 1

Figure S8

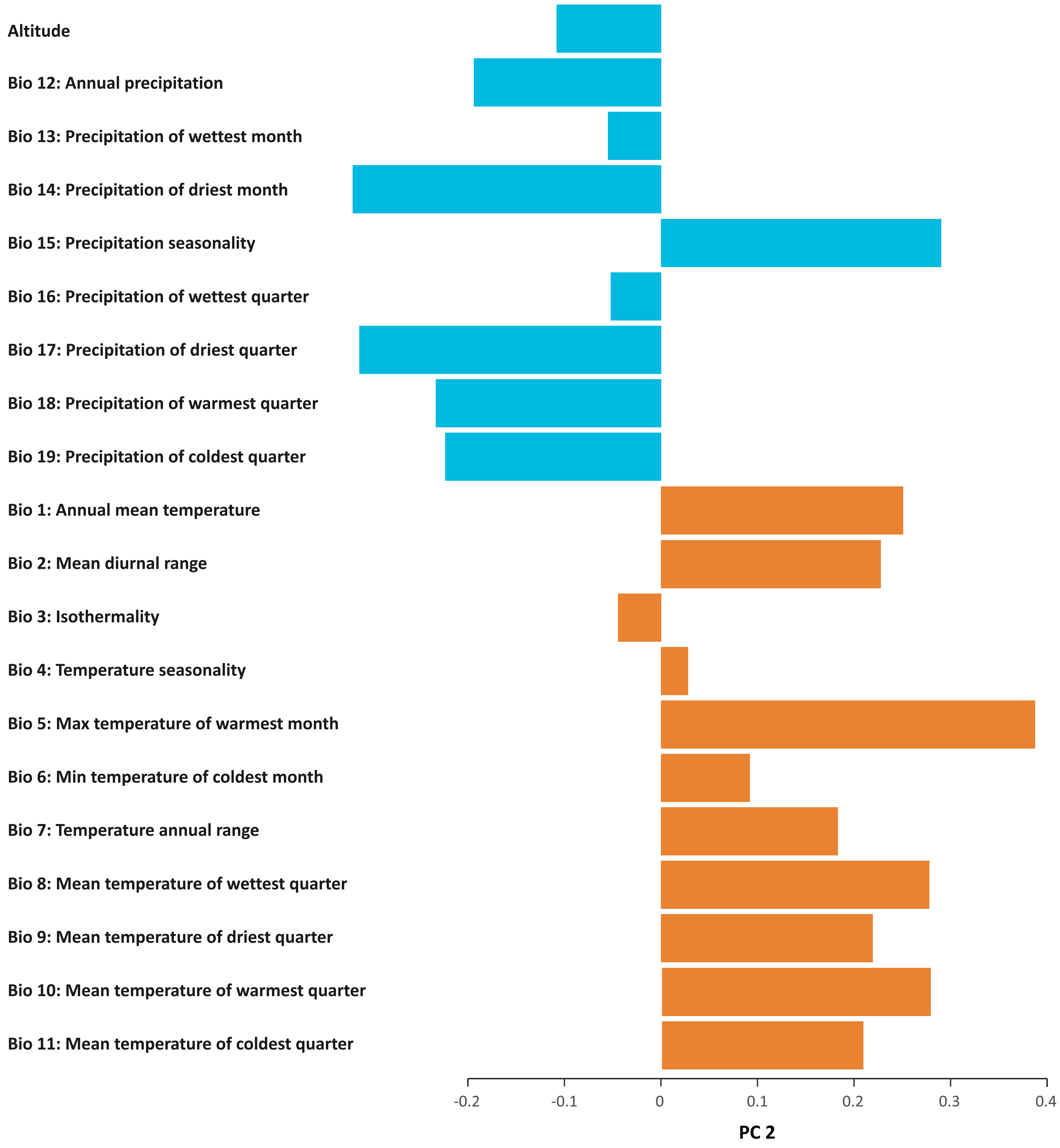
Altitude
Bio 12: Annual precipitation
Bio 13: Precipitation of wettest month
Bio 14: Precipitation of driest month
Bio 15: Precipitation seasonality
Bio 16: Precipitation of wettest quarter
Bio 17: Precipitation of driest quarter
Bio 18: Precipitation of warmest quarter
Bio 19: Precipitation of coldest quarter
Bio 1: Annual mean temperature
Bio 2: Mean diurnal range
Bio 3: Isothermality
Bio 4: Temperature seasonality
Bio 5: Max temperature of warmest month
Bio 6: Min temperature of coldest month
Bio 7: Temperature annual range
Bio 8: Mean temperature of wettest quarter
Bio 9: Mean temperature of driest quarter
Bio 10: Mean temperature of warmest quarter
Bio 11: Mean temperature of coldest quarter
-0.2
-0.1
0
0.1
0.2
0.3
0.4
PC 2

Figure S9

(a)

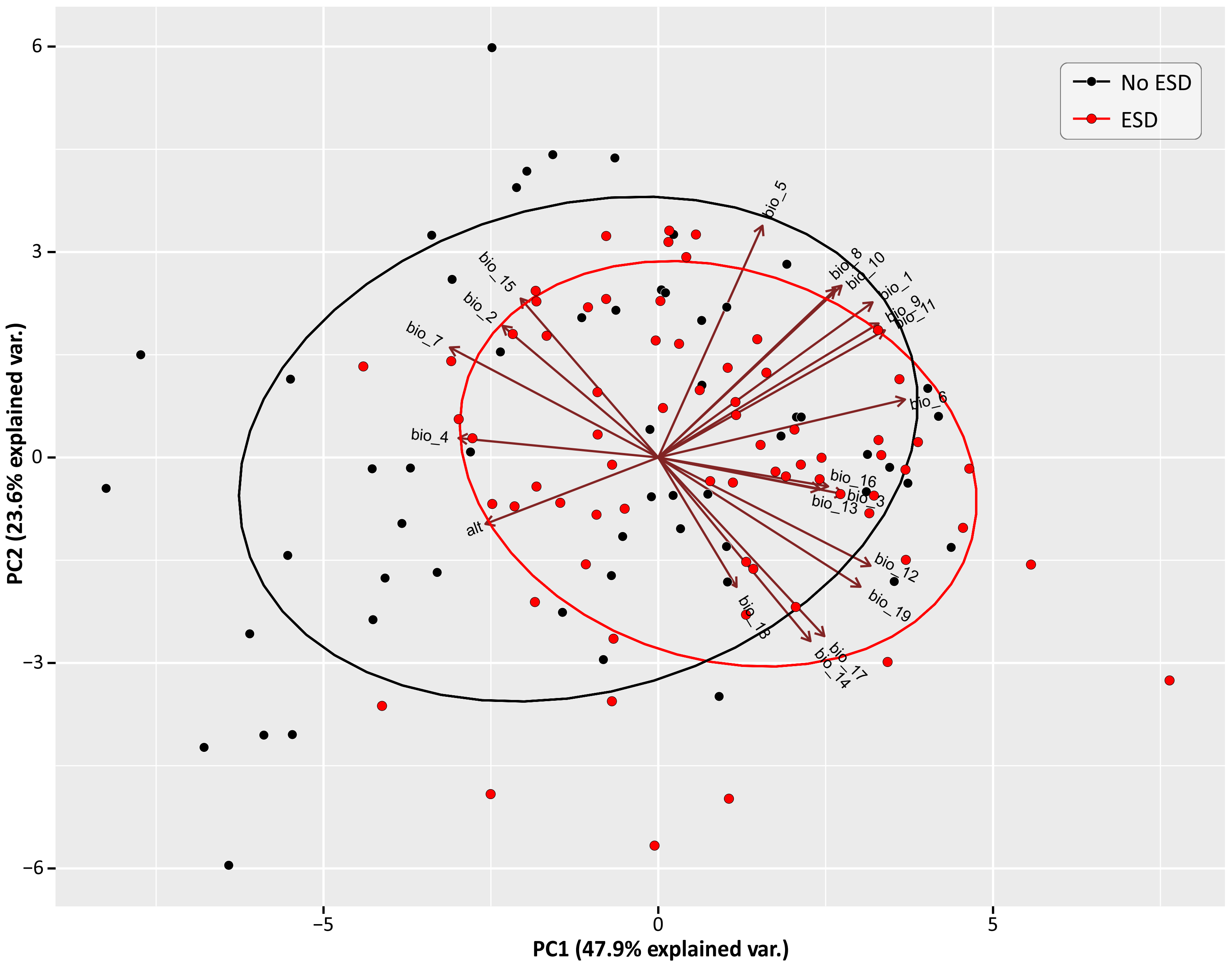

No ESD
ESD
bio_5
bio_8
bio_10
bio_1
bio_9
bio_11
bio_6
bio_15
bio_2
bio_7
bio_4
alt
bio_16
bio_3
bio_13
bio_12
bio_19
bio_18
bio_17
bio_14
6
3
0
−3
−6
−5
0
5
PC1 (47.9% explained var.)
PC2 (23.6% explained var.)

(b)

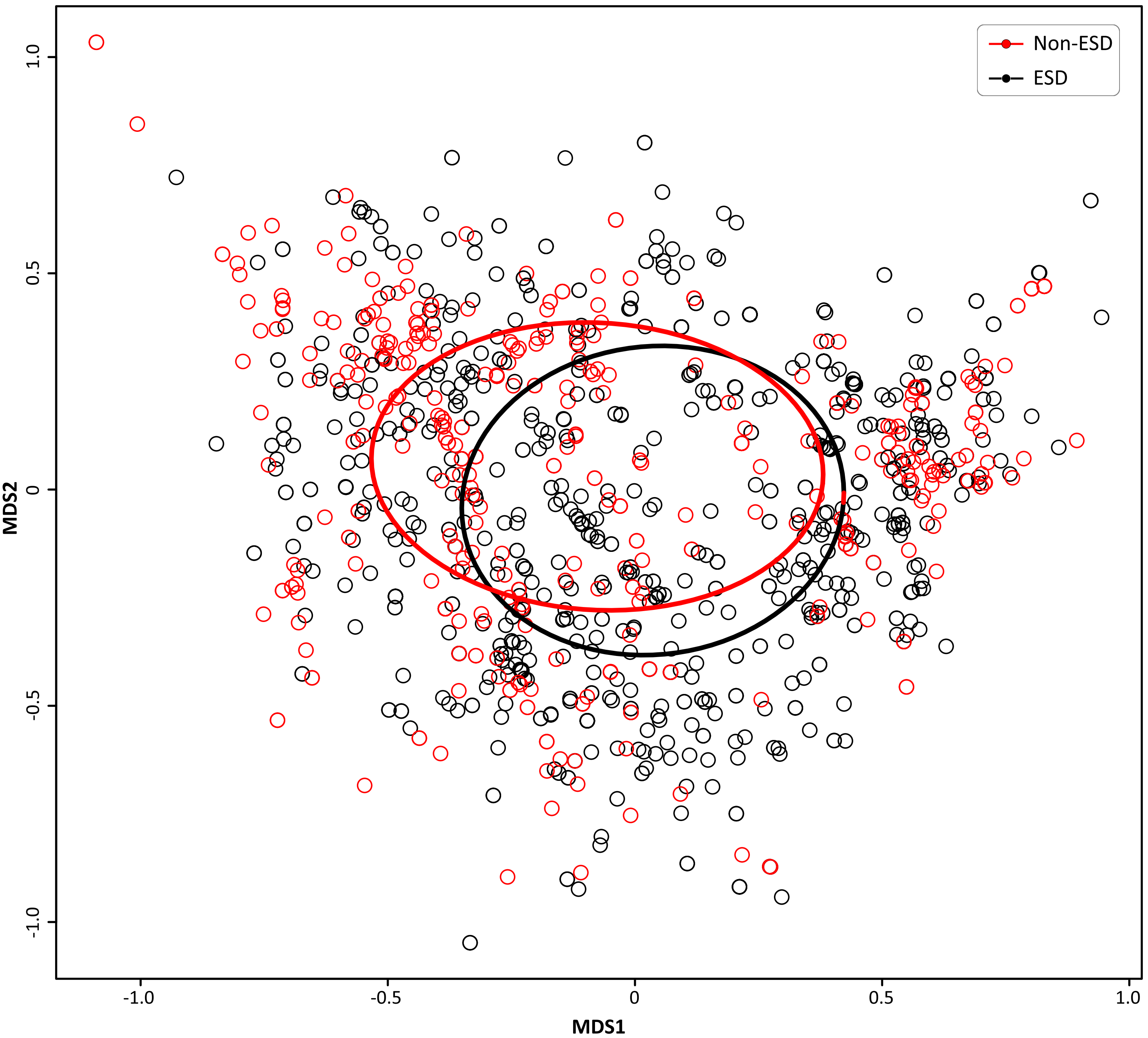

Non-ESD
ESD
1.0
0.5
0
-0.5
-1.0
-1.0
-0.5
0
0.5
1.0
MDS1
MDS2

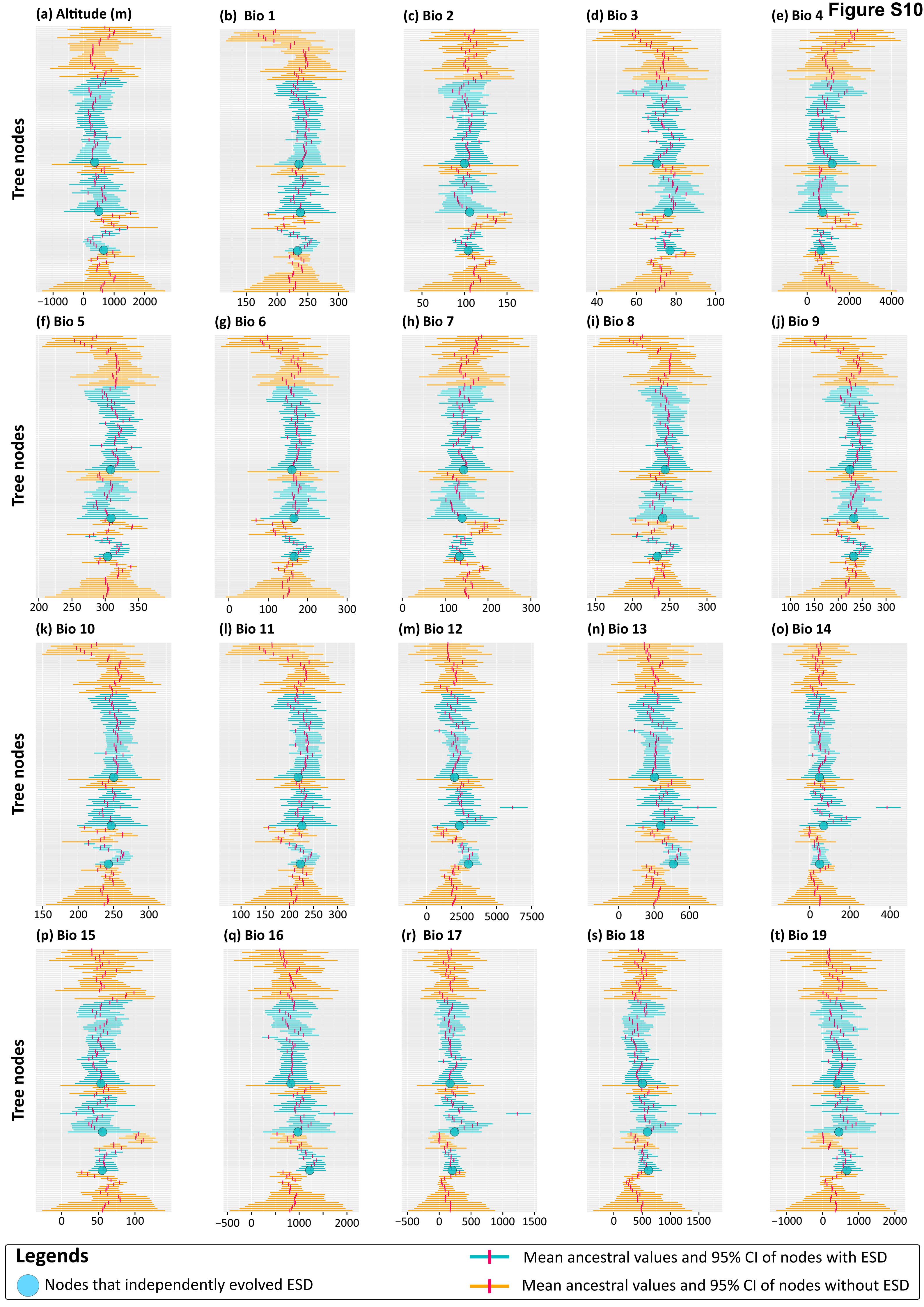
Figure S10
(a) Altitude (m)
(b) Bio 1
(c) Bio 2
(d) Bio 3
(e) Bio 4
(f) Bio 5
(g) Bio 6
(h) Bio 7
(i) Bio 8
(j) Bio 9
(k) Bio 10
(l) Bio 11
(m) Bio 12
(n) Bio 13
(o) Bio 14
(p) Bio 15
(q) Bio 16
(r) Bio 17
(s) Bio 18
(t) Bio 19
Tree nodes
Legends
Nodes that independently evolved ESD
Mean ancestral values and 95% CI of nodes with ESD
Mean ancestral values and 95% CI of nodes without ESD

Figure S11

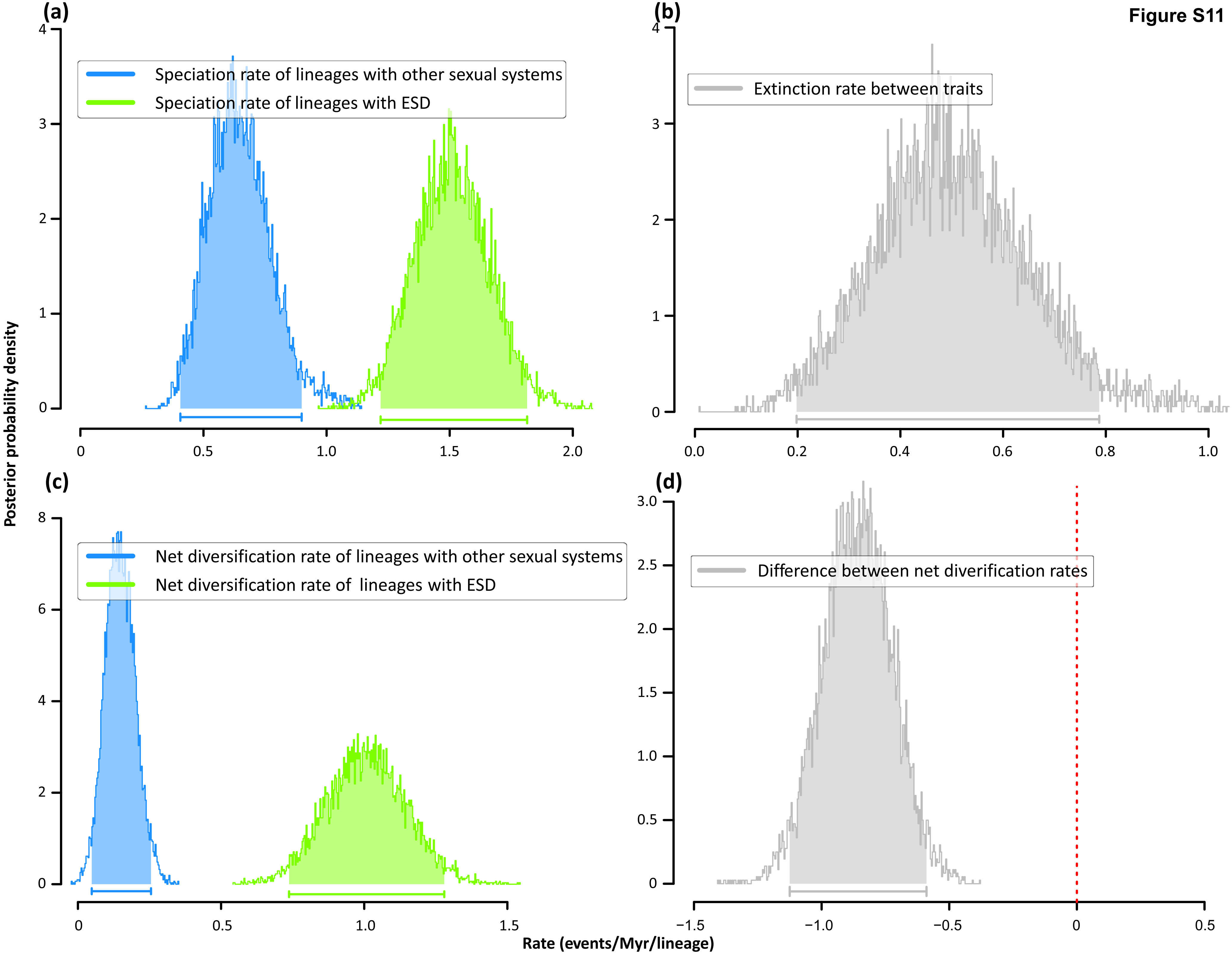
(a)
Speciation rate of lineages with other sexual systems
Speciation rate of lineages with ESD
(b)
Extinction rate between traits
(c)
Net diversification rate of lineages with other sexual systems
Net diversification rate of lineages with ESD
(d)
Difference between net diverification rates
Posterior probability density
Rate (events/Myr/lineage)

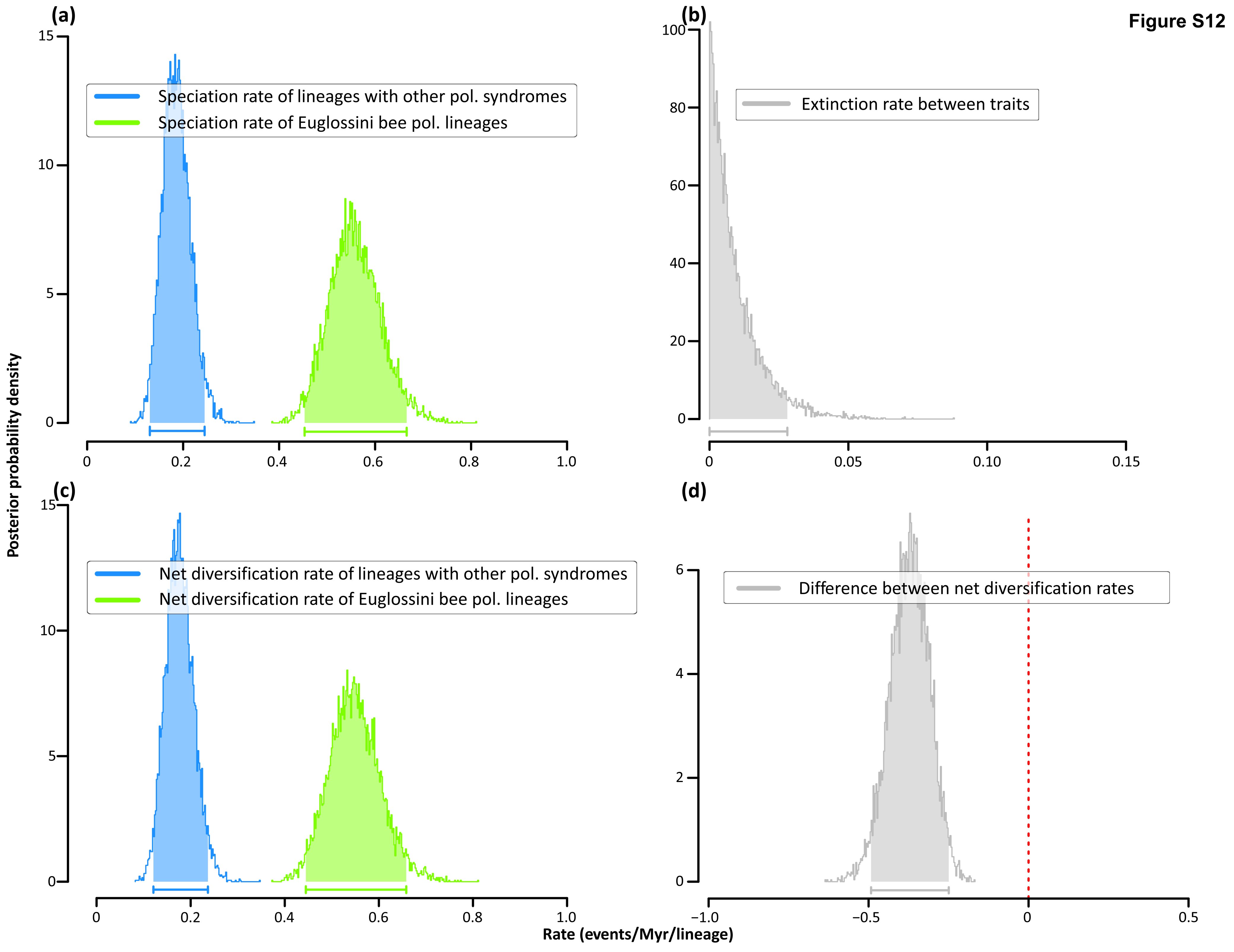
Figure S12
(a)
Speciation rate of lineages with other pol. syndromes
Speciation rate of Euglossini bee pol. lineages
(b)
Extinction rate between traits
(c)
Net diversification rate of lineages with other pol. syndromes
Net diversification rate of Euglossini bee pol. lineages
(d)
Difference between net diversification rates
Posterior probability density
Rate (events/Myr/lineage)
0
5
10
15
0.2
0.4
0.6
0.8
1.0
20
40
60
80
100
0.05
0.10
0.15
2
4
6
−1.0
−0.5
0.5

Figure S13

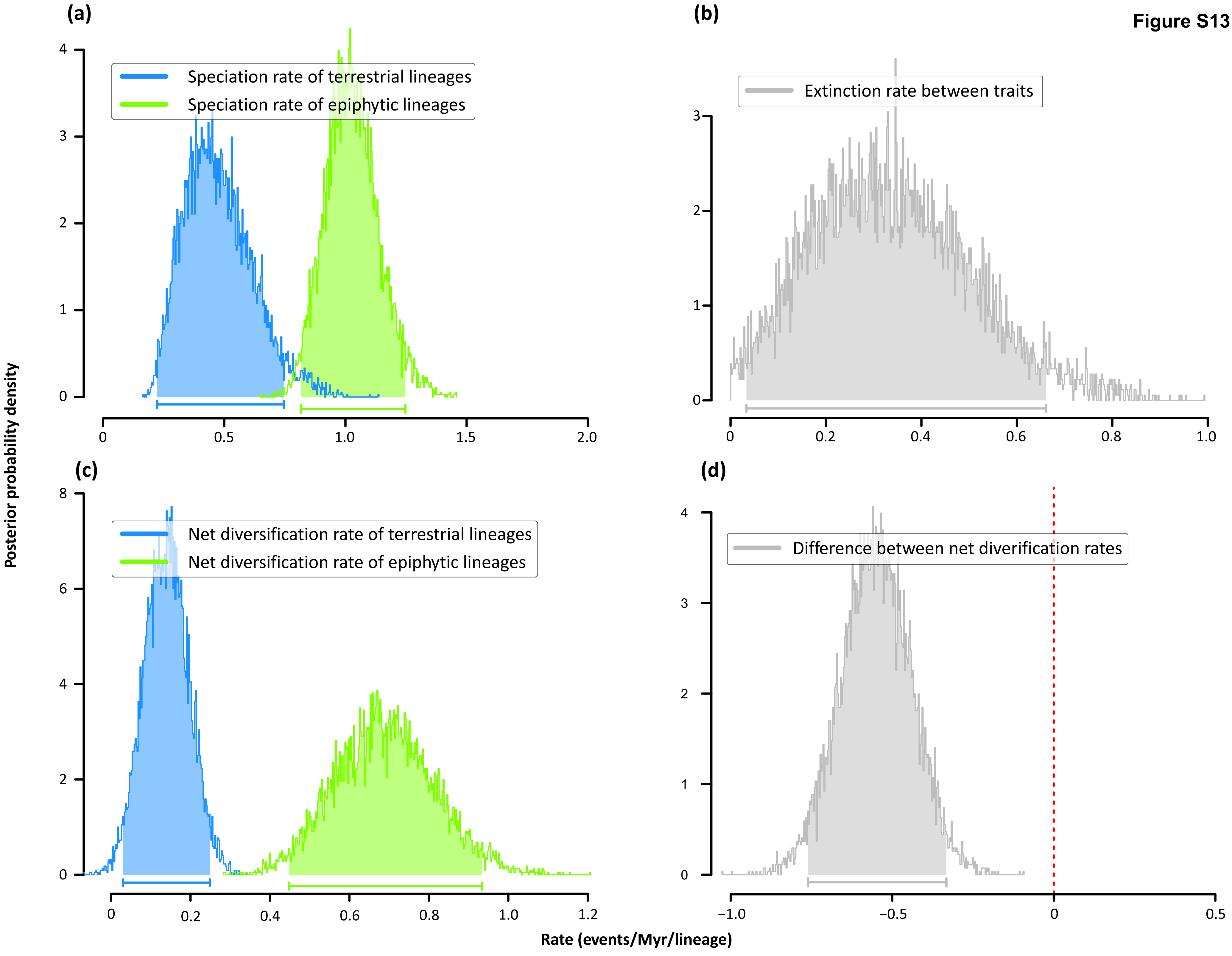

(a)
Speciation rate of terrestrial lineages
Speciation rate of epiphytic lineages
0
1
2
3
4
0
0.5
1.0
1.5
2.0
(b)
Extinction rate between traits
0
1
2
3
0
0.2
0.4
0.6
0.8
1.0
(c)
Net diversification rate of terrestrial lineages
Net diversification rate of epiphytic lineages
0
2
4
6
8
0
0.2
0.4
0.6
0.8
1.0
1.2
(d)
Difference between net diverification rates
0
1
2
3
4
−1.0
−0.5
0
0.5
Posterior probability density
Rate (events/Myr/lineage)

Figure S14

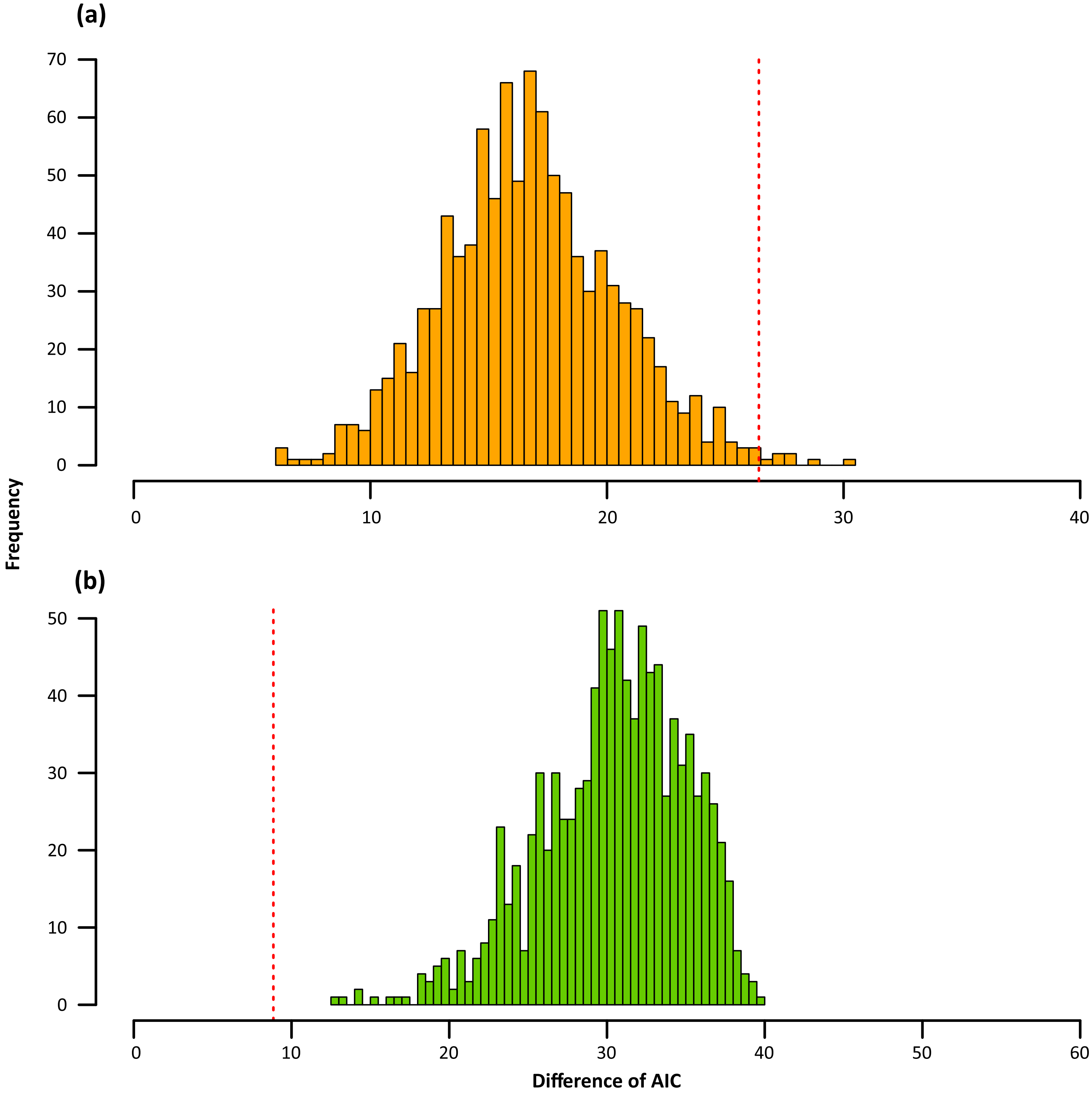

(a)
70
60
50
40
30
20
10
0
0
10
20
30
40
Frequency
(b)
50
40
30
20
10
0
0
10
20
30
40
50
60
Difference of AIC
